# Dynamic Computer-Aided Navigation System in Dentoalveolar Surgery and Maxillary Bone Augmentation in a Dental Setting: A Systematic Review

**DOI:** 10.3390/healthcare13141730

**Published:** 2025-07-17

**Authors:** Federica Di Spirito, Roberta Gasparro, Maria Pia Di Palo, Alessandra Sessa, Francesco Giordano, Iman Rizki, Gianluca Allegretti, Alessia Bramanti

**Affiliations:** 1 Department of Medicine, Surgery and Dentistry, University of Salerno, Via S. Allende, 84081 Baronissi, Italy; a.sessa102@studenti.unisa.it (A.S.); frgiordano@unisa.it (F.G.); i.rizki@studenti.unisa.it (I.R.); dott.allegrettigianluca@gmail.com (G.A.); abramanti@unisa.it (A.B.); 2 Department of Neuroscience, Reproductive Science and Dentistry, University of Naples Federico II, 80131 Naples, Italy; roberta.gasparro@unina.it

**Keywords:** computer-assisted surgeries, patient-reported outcome measures, oral surgical procedures, tooth extraction, third molar, bone regeneration, foreign bodies, maxillary sinus, cysts

## Abstract

**Background**: Dynamic computer-aided navigation systems are a real-time motion tracking technology widely applied in oral implantology and endodontics to enhance precision and reduce complications. However, their reliability, accuracy, and usability in dentoalveolar surgery and maxillary bone augmentation remain underinvestigated. **Methods**: A systematic review following PRISMA guidelines was conducted and registered on PROSPERO (CRD42024610153). PubMed, Scopus, Web of Science, and Cochrane Library databases were searched until October 2024 to retrieve English eligible studies, without restrictions on the publication year, on dynamic computer-assisted navigation systems in dentoalveolar and bone augmentation surgeries. Exclusion criteria were surgery performed without dynamic computer-assisted navigation systems; dental implant placement; endodontic surgery; and maxillo-facial surgery. The outcomes were reliability, accuracy, post-operative course, surgical duration, complications, patient- and clinician-reported usability, acceptability, and satisfaction. Included studies were qualitatively synthetized and judged using dedicated tools for the different study designs. **Results**: Twenty-nine studies with 214 patients were included, showing high reliability in dentoalveolar and bone augmentation surgeries comparable to or superior to freehand surgeries, higher accuracy in dentoalveolar surgery compared to maxillary bone augmentation, and reduced complication rates across all surgeries. While overall surgical duration slightly increased due to technology installation, operative time was reduced in third molar extractions. Patient-reported outcomes were poorly investigated. Clinician-reported outcomes were mixed, but difficulties in the differentiation of soft tissue from hard tissue were recorded, especially in sinus floor elevation. **Conclusions**: Dynamic computer-assisted navigation systems enhance accuracy and safety in dentoalveolar and bone augmentation surgery. Further studies are needed to assess the underinvestigated patient-reported outcomes and standardize protocols.

## 1. Introduction

Dynamic computer-aided navigation systems are motion-tracking-based technologies widely employed across various medical specialties, including neurosurgery, orthopedics, and craniomaxillofacial surgery [1].

These systems utilize a tracking camera and intraoperative guidance to provide real-time, three-dimensional (3D) visualization of surgical instruments on a dedicated computer interface. By continuously aligning and integrating preoperative imaging, patient anatomy, anatomical landmarks, surgical instruments, and optical tracking data, these technologies enhance surgical accuracy throughout the procedure [2,3].

Dynamic computer-aided navigation systems have recently been introduced into dental practice, particularly in dental implant pre-surgical planning and placement [3]. As with static computer-assisted protocols, Cone Beam Computed Tomography (CBCT) and intraoral scanning are utilized during the pre-surgical planning phase in dynamic navigation to generate a virtual surgical plan that optimizes the angulation, position, and depth of implant site preparation. Patient-specific landmarks are identified either by referencing anatomical structures visible on preoperative CBCT images or through the use of a fiducial marker plate supplied by the navigation system [3]. Unlike static systems, dynamic navigation employs an infrared-based tracking mechanism that monitors the drill tip in real time during surgery. As the drill approaches the planned implant site, the system displays cross-sectional images on a dedicated computer interface, allowing the surgeon to dynamically visualize, monitor, and adjust implant placement with high precision throughout the procedure [3]. Dynamic navigation-assisted implant placement has been shown to offer superior accuracy compared to freehand techniques and a modest reduction in angular deviation relative to static computer-aided implant surgery [4]. These systems enhance surgical precision, particularly in complex clinical scenarios involving extensive bone defects, challenging anatomical conditions, or proximity to critical structures, thereby reducing the risk of iatrogenic complications [3].

Building on the same principles, dynamic computer-aided navigation systems are gaining prominence in the field of endodontics, aiding in root canal treatments, re-treatments, and apical surgeries, to facilitate minimally invasive procedures, shorten operative times, and enhance accuracy [5].

Indeed, compared to freehand techniques—where procedural accuracy is highly dependent on the surgeon’s experience—dynamic computer-assisted navigation significantly enhances surgical precision and safety [5]. This technological support not only improves clinical outcomes but also makes complex procedures more accessible to practitioners with varying levels of expertise [6].

While broader implementation in dentoalveolar surgery may be constrained by factors such as the high initial investment, ongoing maintenance costs, and the learning curve associated with system proficiency [7], dynamic navigation has already been employed to support pre-surgical planning and operative phases in challenging scenarios. These include the extraction of displaced or retained third molar roots—particularly in cases with an elevated risk of inferior alveolar nerve (IAN) injury [8], as well as in advanced maxillary bone augmentation procedures such as sinus elevation and distraction osteogenesis [9]. Interestingly, a study by Pera et al. [6] found that inexperienced surgeons using dynamic navigation systems achieved greater accuracy than their more experienced counterparts. The authors hypothesized that this outcome may stem from the greater reliance of less experienced clinicians on the system’s guidance, whereas experienced surgeons may be more inclined to deviate from the digital plan due to their established habits or clinical confidence [6].

However, while the literature offers robust evidence supporting the accuracy and clinical benefits of dynamic navigation in dental implantology and endodontics [10], its application in dentoalveolar and maxillary bone augmentation procedures remains underexplored, with current evidence limited to isolated studies. These preliminary reports do not yet provide a comprehensive understanding of the system’s advantages, limitations, and clinical potential in these specific surgical domains, making it difficult to draw evidence-based conclusions or clinical guidelines. The present systematic review is therefore necessary to consolidate current evidence, identify existing knowledge gaps, and provide clinicians and researchers with a structured overview of the accuracy, safety, and patient and clinical outcomes of dynamic navigation in dentoalveolar and maxillary bone augmentation.

To address this knowledge gap, the present systematic review primarily aims to evaluate the reliability and accuracy of dynamic computer-assisted navigation systems in dentoalveolar and maxillary bone augmentation surgeries. Secondarily, it seeks to assess the postoperative course, surgical duration, complication rates and types, and patient- and clinician-reported outcomes related to usability, acceptability, and satisfaction and compare these outcomes with freehand and static computer-assisted or robotic computer-assisted methods.

## 2. Materials and Methods

### 2.1. Study Protocol

The study protocol, registered on 12 November 2024, on the International Prospective Register of Systematic Reviews (PROSPERO) with the code number CRD42024610153, was drafted prior to beginning the search in the scientific literature, data extraction, and analysis process, in compliance with the Preferred Reporting Items for Systematic Reviews and Meta-Analyses (PRISMA) statement [11].

The research question “What are the reliability, accuracy, patient- and clinician-reported usability, acceptability, and satisfaction of dynamic computer-assisted navigation systems in dentoalveolar surgery and maxillary bone augmentation?” was based on the PICOs model (Population–Intervention–Comparison–Outcome Studies) [12] as follows:

- (P) Population: subjects who have undergone dentoalveolar surgery and maxillary bone augmentation with dynamic computer-assisted navigation systems;
- (I) Intervention: dentoalveolar surgery and maxillary bone augmentation with dynamic computer-assisted navigation systems (any);
- (C) Comparison: dentoalveolar surgery and maxillary bone augmentation freehand, or with full or half static computer-assisted, or robotic computer-assisted methods;
- (O) Outcome(s):
  ○ Primary outcome(s): reliability and accuracy of dynamic computer-assisted navigation systems in dentoalveolar surgery and maxillary bone augmentation (measured as angle deviation, entry deviation, depth deviation, linear lateral deviation);
  ○ Secondary outcome(s): post-operative course, surgical duration, complication rates, and type of dentoalveolar surgery and maxillary bone augmentation performed with any dynamic computer-assisted navigation system; patient- and clinician-reported usability, acceptability, and satisfaction.

### 2.2. Search Strategy

The electronic search was performed independently by two reviewers (F.D.S., A.S.) on PubMed/MEDLINE, Scopus, Web of Science (WOS), and Cochrane Library databases until 28 October 2024 to retrieve studies in the English language without restrictions on the year of publication using the following keywords combined with Boolean operators: (“dynamic computer-aided surgery” OR “real-time navigation” OR “real-time surgery” OR “navigation system” OR “dynamic guided surgery” OR “dynamic computer-assisted surgery” OR “dynamic navigation” OR “navigation surgery”) AND (“oral surgery” OR dentistry) AND (dynamic OR navigation).

The filter applied to refine the search was “English” in the PubMed/MEDLINE, Scopus, WOS, and Cochrane Library databases.

For each database, the full search strategy was provided in Supplementary File S1.

The lists of references of the included studies were independently screened by the same two reviewers (F.D.S., A.S.) to retrieve additional potential records manually.

### 2.3. Study Selection and Eligibility Criteria

Two reviewers (F.D.S., A.S.) independently recorded the collected citations, eliminated the duplicates, screened the obtained title-abstracts, and if the abstracts of the potentially relevant records were ambiguous, the same reviewers (F.D.S., A.S.) read the full texts independently. To assess inter-rater reliability between the two independent reviewers, Cohen’s kappa statistic was calculated for the study selection and the risk of bias assessment using the IBM SPSS Statistics for Windows (version 2017; IBM Corp., Armonk, NY, USA). A third reviewer (A.B.) was engaged to resolve any issues of disagreement through discussion.

The same study selection process was conducted for the manual search in the lists of references of included studies.

The Mendeley Reference Manager tool was used to tabulate all references of the included studies through both the electronic and the manual search.

The authors of the screened studies were contacted if the full text sought for retrieval was unavailable.

Inclusion criteria were case reports, case series, case–control, cross-sectional, observational studies, and randomized and non-randomized clinical trials in the English language and without restrictions on the year of publication, concerning dentoalveolar surgery and maxillary bone augmentation with dynamic computer-assisted navigation systems (any). No restrictions regarding the publication year, sample size, age, and gender were applied.

Exclusion criteria were preclinical and in vitro studies, reviews (any type), conference papers, books or chapters, and oral communications; dental implant (endo/juxta-osseous, zygomatic, pterygoid, orthodontic) placement surgery; dentoalveolar surgery and maxillary bone augmentation without dynamic computer-assisted navigation systems; endodontic surgery; maxillo-facial surgery; and others.

### 2.4. Data Extraction and Collection

The data from the included studies were independently extracted and collected by two reviewers (F.D.S., A.S.) in a standardized form for data extraction compliant with the proposed models for intervention reviews of nonrandomized and randomized clinical trials [13]. A third reviewer (A.B.) was engaged to resolve any issues of disagreement through discussion.

The data from the included studies extracted and collected were

- Study characteristics: first author, year, journal, reference, study design, quality assessment, funding;
- Population characteristics: sample size, gender ratio, mean/range age, comorbidities, ongoing pharmacological treatment, dentition status;
- Oral surgery characteristics: type of pre-implant/dentoalveolar surgery, surgery step performed with dynamic computer-assisted navigation (half static computer-assisted or robotic computer-assisted methods for the comparison), location, other characteristics;
- Intervention/Comparison characteristics:
  - Intervention dynamic computer-assisted navigation characteristics: dental impression technique, radiographic imaging, guidance method for imaging (if any), planning software, navigation software, navigation system, guidance method for navigation (if any);
  - Comparison: dental impression technique, radiographic imaging, guidance method for imaging (if any), planning software, computer/robotic-assisted software, computer/robotic-assisted system, guidance method for navigation (if any);
- Primary outcome(s): angle deviation, entry deviation, depth deviation, linear lateral deviation;
- Secondary outcome(s): surgical duration, post-operative course, complication(s), rate and type of dentoalveolar surgery and maxillary bone augmentation, follow-up; patient- and clinician-reported usability, acceptability, and satisfaction.

### 2.5. Data Synthesis

The data extracted and collected from the included studies were qualitatively synthesized, focusing on the population, oral surgery, and intervention/comparison characteristics, as well as on the primary and secondary outcomes, by performing a descriptive statistical analysis using the Microsoft Excel Software 2019 (Microsoft Corporation, Redmond, WA, USA) to

- Estimate the reliability and the accuracy of dynamic computer-assisted navigation systems in dentoalveolar surgery and maxillary bone augmentation;
- Compare the reliability and the accuracy of dynamic computer-assisted navigation systems in dentoalveolar surgery and maxillary bone augmentation vs. dentoalveolar surgery and maxillary bone augmentation freehand vs. full or half static computer-assisted vs. robotic computer-assisted methods;
- Estimate the patient-reported and clinician-reported usability, acceptability, and satisfaction of dynamic computer-assisted navigation systems in dentoalveolar surgery and maxillary bone augmentation;
- Compare the patient-reported and clinician-reported usability, acceptability, and satisfaction of dynamic computer-assisted navigation systems in dentoalveolar surgery and maxillary bone augmentation vs. dentoalveolar surgery and maxillary bone augmentation freehand vs. full or half static computer-assisted vs. robotic computer-assisted methods;
- Estimate and compare the reliability and the accuracy of dynamic computer-assisted navigation systems in dentoalveolar surgery and maxillary bone augmentation among different surgeries performed through dynamic computer-assisted navigation systems.

### 2.6. Quality Assessment

The studies included in the present systematic review were qualitatively judged by two independent reviewers (F.D.S., A.S.) using dedicated tools for the different study designs, accessed on 8 November 2024, as follows: the Risk of Bias in Nonrandomized (ROBINS-I) and the revised Cochrane Risk of Bias for Randomized (RoB-II) Studies of Interventions for nonrandomized and randomized studies, respectively (freely available on https://www.riskofbias.info/welcome/home; https://sites.google.com/site/riskofbiastool/welcome/rob-2-0-tool?authuser=0, accessed on 8 November 2024); the Johanna Briggs Institute (JBI) for case series and case report studies (freely available on https://jbi.global/critical-appraisal-tools, accessed on 8 November 2024). A third reviewer (A.B.) was engaged to resolve any issues of disagreement through discussion.

## 3. Results

### 3.1. Study Selection

The electronic searches from the databases retrieved 1200 records, 590 from MEDLINE/PubMed, 473 from Web of Science, 128 from Scopus, and 9 from Cochrane Library; 361 duplicates were identified and removed. The remaining 839 titles/abstracts were screened and 792 were excluded because they were not eligible for the scope of the present systematic review. Of the remaining 47 records, the full-texts were screened. Taking into consideration the eligibility criteria, 21 records were excluded for the following reasons: 11 records were not dentoalveolar surgery and maxillary bone augmentation, 5 were not on dynamic computer-assisted navigation systems, 2 were on maxillo-facial surgery, 1 showed the absence or inability to extract data related to oral surgery, and 1 was a review. The authors were contacted to obtain the full texts, but due to the missing responses, three records were excluded.

A total of 23 studies [14,15,16,17,18,19,20,21,22,23,24,25,26,27,28,29,30,31,32,33,34,35,36] were included in the present systematic review.

Additionally, a total of 475 records that were manually retrieved through the reference lists of the 23 articles included in this systematic review [14,15,16,17,18,19,20,21,22,23,24,25,26,27,28,29,30,31,32,33,34,35,36], were examined. Of these, 85 were removed because of duplicates, 338 titles/abstracts were not in compliance with the scope of the present systematic review, and the full-texts of the remaining 52 articles were screened. Taking into consideration the eligibility criteria, 43 records were excluded for the following reasons: 17 were not dentoalveolar surgery and maxillary bone augmentation, 9 were reviews, 7 were not on dynamic computer-assisted navigation systems, 5 were on maxillo-facial surgery, 4 were in vitro studies, and 1 was oral communication. The authors were contacted to obtain the full texts, but due to the missing responses, two records were excluded.

A total of six studies [37,38,39,40,41,42] found from the manual search were included in the present systematic review.

The inter-rater reliability with Cohen’s kappa was 0.79 for study selection.

**Figure 1:**
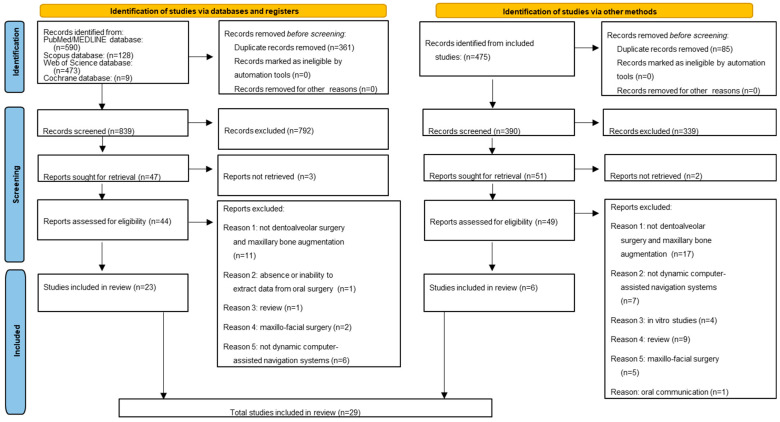
Flow diagram of the PRISMA 2020 statement: the study selection from the electronic and manual search.

Finally, 29 articles [14,15,16,17,18,19,20,21,22,23,24,25,26,27,28,29,30,31,32,33,34,35,36,37,38,39,40,41,42] on dentoalveolar surgery and maxillary bone augmentation with dynamic computer-assisted navigation systems were considered in the present systematic review (Figure 1).

### 3.2. Study Characteristics and Qualitative Synthesis

Of the 29 studies included and synthesized in Table 1 (studies, population, and intervention characteristics) and in Table 2 (primary and secondary outcomes), 20 were case reports [15,16,17,19,21,22,24,26,27,28,29,30,31,32,33,35,36,37,40,41], 5 were case series [14,20,25,34,38], 2 were RCTs [18,42], and 2 were non-randomized studies [23,39] involving a total of 214 participants, 105 females and 109 males, between 7 and 86 years with a mean age of 32.59 [14,15,16,17,18,19,20,21,22,23,24,25,26,27,28,29,30,31,32,33,34,35,36,37,38,39,40,41,42].

Dentoalveolar surgeries performed through Dynamic Computer Navigation were described in 12 studies [14,15,17,18,20,25,30,33,35,37,38,42] involving 131 patients, 69 males and 62 females between 7 and 56 years old (mean age: 21.34), who underwent tooth extraction: third molar extraction surgeries (*n* = 96; 44.86%) [15,18,25,38], supernumerary tooth extraction surgeries (*n* = 21; 9.81%) [17,20,35,37,42], teeth extraction surgeries (*n* = 2; 0.93%) [30,33], and coronectomy of third molar surgeries (*n* = 12; 5.61%) [14].

Other dentoalveolar surgeries were described in 13 studies [16,20,21,24,27,28,29,31,32,34,36,40,41] involving 18 patients (8.41%), 8 males and 10 females between 13 and 86 years, with a mean age of 32.27. Six patients (2.80%) underwent the removal of a foreign body for broken dental needles (*n* = 2; 33.33% of the removal of foreign body surgeries) [29,31], high-speed fissure bur (*n* = 1; 16.67%) [24], compound resins used for a tooth filling (*n* = 1; 16.67%) [27], broken dental instrument (*n* = 1; 16.67%) [28]; buckshot (*n* = 1; 16.67%) [41]; seven subjects underwent dental implant removal surgery (3.27%) [20,34]; two patients cyst removal surgery (0.93%) [36,40]; one patient underwent bone graft fixing screw removal surgery (0.47%) [32]; one patient underwent sequestrectomy for stage 2 medication-related osteonecrosis of the jaws (0.47%) [16]; and one patient underwent osteoplasty surgery before the implant placement (0.47%) [21].

Maxillary bone augmentation surgeries performed through Dynamic Computer Navigation were described in five studies [19,22,23,26,39] involving 65 (30.38%) patients, 32 males and 33 females between 27 and 78 years, with a mean age of 49.49. Sixty-four patients underwent sinus elevation surgeries (29.91%), in particular lateral access (*n* = 1; 1.56%) of the sinus elevation surgeries, transcrestal (*n* = 28; 43.75%), and lateral and transcrestal (*n* = 35; 54.68%) [19,23,39]. One patient underwent a “Sandwich” procedure (0.47%) [26]. There are no available data for sample sizes that underwent the autogenous bone ring technique [22].

**Figure 2:**
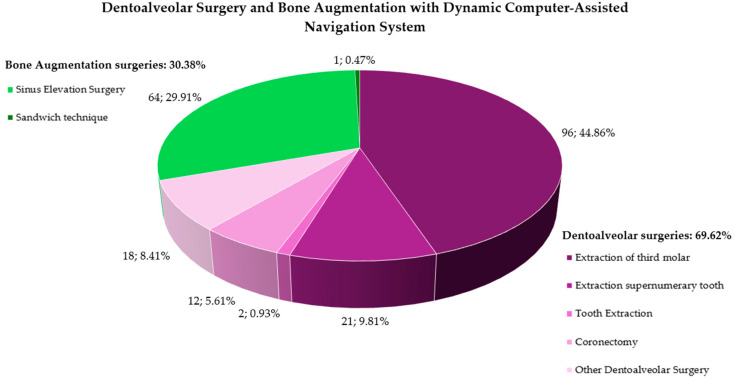
Percentage of patients who underwent dentoalveolar surgery (69.62%) and bone augmentation (30.38%) with the dynamic computer-assisted navigation system.

Surgical procedures described in the included studies are depicted in Figure 2.

### 3.3. Dentoalveolar Surgeries

#### 3.3.1. Tooth Extraction

Two studies reported the extraction of a tooth root in a 55-year-old partially edentulous patient (0.93%) [33] and the extraction of two molars in a 16-year-old dentulous patient (0.93%) [30].

The initial radiographic evaluations were carried out using OPT [30] and CBCT [30,33].

One tooth root was located on the right mandibular region near the IAN [33] and two molars were located at the maxillary sinus, both with alveolar trauma and bilateral condyle fractures [30].

The Dynamic Computer Navigation devices used were the BrainLAB^®^ navigation system [33]; the VectoriVision2^®^ navigation system [30], which helped with an all-in-one splint [33]; and the Z-touch laser pointer [30].

The surgery included extracting the teeth one by one [30] and performing osteotomy [33] and lasted 50 min [30] and 55 min, with the resolution of the discomfort in the right mandibular region [33].

One study recorded a deviation of 0.5 mm at the right first mandibular molar [33].

#### 3.3.2. Extraction of Supernumerary Tooth

Five studies reported the extraction of supernumerary teeth in a total of 21 (9.81%) patients between 7 and 29 years old, with a mean age of 10.04 [17,20,35,37,42].

Only two of the five studies carried out a dental impression technique using a thermoplastic clip [35] and a modified occlusal registration [37].

The initial radiographic imaging evaluations were performed using CBCT [17,20,35,37,42] implemented with a preformed splint [20], a fiducial apparatus [35], radiopaque spatial markers [17] and BrainLAB^®^ spheres [37,42].

One tooth was on the lingual side of the mandible between the left second premolar and the left first molar [17]; two teeth were on the right side of the mandible in proximity to the region of the mental foramen [20]; two teeth were in the upper central region underneath the nasal cavity [20,35]; one tooth was in the right second premolar region in proximity to the maxillary sinus [35]; two teeth were in the upper central region in proximity to the unerupted canine and the root of the lateral incisor [37]; and sixteen teeth were in the upper central region close to the adjacent teeth, the nasal floor, the maxillary sinus, and the nasopalatine nerve canal [42].

The Dynamic Computer Navigation devices used were Brain LAB iPLAN^®^ planning software (version 2.1) [42] and the Brain LAB ENT/CMF navigation system [20,37], and DCARER^®^ planning software and DHC-D12 navigation system [17], which helped with a preformed splint [20], headband, and modified occlusal registration [37,42]; visible fiducial landmarks [35]; and infrared light emitted on spatial markers [17] to record mucosal and bony reference points.

Three studies described the use of Dynamic Computer Navigation performing the osteotomy surgery step [17,35,42], confirming the position of the supernumerary tooth [42]; in another one, the Dynamic Computer Navigation was used to perform the extraction procedure [37]. In one study [20], data was not available.

The surgeries lasted a minimum of 10 min [42] to a maximum of 30 min [37].

One study reported complications after the intervention; the patient self-reported mild pain after the intervention, with resolution at two weeks and expressed positive feedback about the dynamic computer navigation system [17].

Two studies reported the accuracy value of Dynamic Computer Navigation devices. The first study reported a registration accuracy of 0.25 mm, and the accuracy of the landmarks ranged from 0.6 to 1.4 mm [37]. The second study reported that all 16 teeth were exposed at the planned access point (entry deviation); the extra removed bone was 0.0 mm in in length (maximum deviation 4.0 mm) (depth deviation) and 0.0 mm in width (maximum deviation 2.0 mm) (linear lateral deviation).

One study reported the patient’s pain, measured the second day after surgery using VAS (1.37 ± 0.16) [42].

#### 3.3.3. Extraction of Third Molar

Four studies reported the extraction of third molars in a total of 96 (44.86%) dentulous patients between 18 and 56 years old, with a mean age of 24.34 [15,18,25,38].

One study reported the subject’s comorbidities of a 56-year-old subject who had a history of hepatitis B and hypertension [15]; in the other three studies, subjects had no comorbidities [18,25,38].

The initial radiographic imaging evaluations were performed using CBCT [15,18,25,38] aided by a U-shaped tube filled with silicone rubber [18], a resin radiopaque occlusal splint [15], and a marker plate [25].

A total of 83 teeth were in the mandibular third molar region in proximity to the IAN [18,25] and 80 of them were adjacent to the second molar in a deep horizontal orientation [18]; 7 teeth were located in the sublingual space; 4 teeth were located in pterygomandibular space; 1 tooth was located in the lateral pharyngeal space [38]; and one tooth was located in the mandibular third molar region on the sublingual space above the mylohyoid line [15].

The Dynamic Computer Navigation devices used were the Mimics 21.0^®^ planning system (Materialise NV, Leuven, Belgium) [18], the BrainLAB^®^ Kolibri ENT system (BrainLAB AG, Munich, Germany) as a navigation system [38], the BrainLAB^®^ Curve navigation system (BrainLAB AG, Munich, Germany) [15], and ImplaNAV (BresMedical Pty. Ltd., Ingleburn, New South Wales, Australia) as the navigation system [25], which helped with silicone rubber fixed with dental resin [18], a Z-touch laser pointer (BrainLAB AG, Munich, Germany) [38], a resin radiopaque occlusal splint [15], and a marker plate, keeping attached the patient reference tool [25].

The surgery steps included osteotomy [18,25] and ostectomy [18] or clamping [38] before the extraction process [18,38] and lasted a minimum of 15 min [38] to a maximum of 37 ± 5 min [18].

One study reported complications after the intervention, and pain showed resolution within the first week [38].

#### 3.3.4. Coronectomy of the Third Molar

One study [14] of the twenty-nine studies included [14,15,16,17,18,19,20,21,22,23,24,25,26,27,28,29,30,31,32,33,34,35,36,37,38,39,40,41,42] described the coronectomy procedure of mandibular third molars in a total of 12 patients (5.61%), 5 males, and 7 females between 24 and 32 years old, with a mean age of 28.67 [14].

The study carried out a dental impression technique using a digital intraoral scanner [14].

The initial radiographic evaluations were performed using CBCT [14].

All the teeth were in proximity to the IAN [14].

The Dynamic Computer Navigation devices used were GeoMagicTM Studio 12 software^®^ as planning software; Dcarer^®^, as navigation software, helped with alignment grooves [14].

The surgery included trimming the remaining tooth until it reached the ideal depth [14] and lasted from 30 to 40 min [14].

The root mean square deviation was 0.69 ± 0.21 mm, maximum 1.45 ± 0.83/1.87 ± 0.63 [14].

The follow-up was positive with no infections, pulpitis, dry socket, and postoperative root eruption at three months [14].

The clinicians’ feedback conveys that the current navigation system was not equipped to support irregular 3D boundaries when performing tooth sectioning [14].

#### 3.3.5. Removal of Foreign Bodies

Six studies [24,27,28,29,31,41] of the twenty-nine studies included [14,15,16,17,18,19,20,21,22,23,24,25,26,27,28,29,30,31,32,33,34,35,36,37,38,39,40,41,42] described the removal procedures of foreign bodies in soft [29,31,41] and bone tissues [24,27,28] and incorporated a total of six participants (2.80%), five females and one male, between 13 and 65 years old, with a mean age of 36 [24,27,28,29,31,41].

Five studies reported the presence of foreign bodies for idiopathic reasons: broken dental needle (*n* = 2, 33.33%) [29,31], a high-speed fissure bur (*n* = 1, 16.67%) [24], compound resins used for tooth filling (*n* = 1, 16.67%) [27], and a broken dental instrument (*n* = 1, 16.67%) [28]; in contrast, in one study it was reported that the foreign body was buckshot (*n* = 1, 16.67%) [41].

The radiographic evaluations were performed using OPT [24,28], CBCT [24,27,28,29,31,41], face scans [27], customized interocclusal splints [28,31], and buckshots themselves [41].

One was in the medial pterygoid muscle [31], one in the mandibular lingual soft tissue [24], one in the posterior mandible bone tissue near to the IAN [27], one in the pterygomandibular region medial to the mandibular ramus [29], one in the right mandibular first premolar region near the IAN [28], and twenty-four buckshots in many regions of the face and neck: soft tissues of the left buccal sulcus (*n* = 7), soft tissues of the labial sulcus (*n* = 3), soft tissues of the inferior mandible margin (*n* = 3), soft tissues of the submental and submandibular regions (*n* = 4), mandible (*n* = 3), floor of the mouth (*n* = 2), neck (*n* = 1), and maxillary alveolar mucosa (*n* = 1) [41].

The Dynamic Computer Navigation devices used were the iPLAN BrainLAB^®^ planning software (BrainLAB AG, Munich, Germany) [27], ENT 2.2.2. planning software [29], Medttonic navigation Inc.^®^ planning software (Medtronic Navigation, Inc., Louisville, CO, USA) [28], Accu-Navi-A plan software and navigation system (AccuNavi-A, UEG Medical, Shanghai, China) [24], Medtronic^®^ StealthStation^TM^ S7 navigation system [29], Medtronic^®^ StealthStation^TM^ S8 navigation system [31], BrainLAB^®^ navigation software (BrainLAB AG, Munich, Germany) [27], and VectorVision2 Navigation System BrainLAB^®^ navigation system [27,41], which helped with the customized mandible reference frame [24], the self-curing acrylic resin open splint [27], the tracker EM for soft tissue landmarks and hard tissue points [28], and a headband and a Z-touch laser scanner [41]. One study reported the accuracy (0.8 mm) of the Dynamic Computer Navigation devices [24].

One study reported registration system accuracy of 0.8 mm [27].

One study recorded 60 min of surgery [24]. In one study, the follow-up after 1 month had satisfactory wound healing and mouth opening without complications [24].

#### 3.3.6. Dental Implant Removal Surgeries

Two studies [20,34] of the twenty-nine studies included [14,15,16,17,18,19,20,21,22,23,24,25,26,27,28,29,30,31,32,33,34,35,36,37,38,39,40,41,42] described surgeries of dental implant removal and incorporated a total of seven patients, five males and two females, between 55 and 76 years old (3.27%), with a mean age of 64.54 [20,34].

The initial radiographic evaluation was performed using CBCT [34].

A total of three dental implants were located at the anterior maxilla region, two dental implants were located at the molar mandible region, one dental implant was located at the molar region and the maxillary sinus [34], and two dental implants were located at the upper central incisors region in contact with the bilateral cortical bone, almost horizontally [20].

One patient manifested peri-implantitis, four implants were fractured, one patient manifested peri-implantitis and the migration of the dental implant into the maxillary sinus, and five dental implants were next to adjacent teeth [34].

The Dynamic Computer Navigation devices used were the iPLAN BrainLAB^®^ navigation system [20], which helped with reference points fixed on the forehead [34].

The surgery included the removal of the alveolar bone [34].

#### 3.3.7. Cyst Removal

Two studies [36,40] of the twenty-nine studies included [14,15,16,17,18,19,20,21,22,23,24,25,26,27,28,29,30,31,32,33,34,35,36,37,38,39,40,41,42] described surgeries for the removal of residual lesions and incorporated a total of two dentulous male patients between 31 and 38 years old, with a mean age of 34.5 (0.93%) [36,40].

The initial radiographic evaluations were performed using OPT [40], CBCT [36,40] aided by radiopaque markers [36], and numbered landmarks (10 gutta-percha markers) [40].

One cyst was in the right mandibular second and third molar region near the IAN [40] and one cyst was in the maxillary left second and third molar region, including the medial buccal root of the left upper second molar, into one-third of the cavity of the cyst [36].

The Dynamic Computer Navigation devices used were Kick Navigation system, BrainLAB^®^ (BrainLAB AG, Munich, Germany) as planning software and Kick Navigation System BrainLAB^®^ as a navigation system [40], which helped with AR glasses, HoloLens [36] and numbered landmarks [40].

The surgery included removing the cyst [36,40] and the resection of the apex of the medial buccal root of the left upper second molar [36]. One study recorded a quantitative accuracy in root mean square between 3 and 6 mm [36].

#### 3.3.8. Removing Bone Graft Fixing Screws

One study [32] of the 29 studies included [14,15,16,17,18,19,20,21,22,23,24,25,26,27,28,29,30,31,32,33,34,35,36,37,38,39,40,41,42] described the removal of bone graft fixing screws on a 24-year-old partially dentulous female patient (0.47%) [32].

The initial radiographic evaluations were performed using CBCT aided by a U-shaped registration mold consisting of special fiducial markers [32].

The fixing screws were located at the maxillary left first molar and between the first and second premolar regions [32].

The Dynamic Computer Navigation devices used were IGI software^®^ (IGI Technology Inc., San Francisco, CA, USA) as planning and navigation software and a specialized trackable handpiece of the IGI system as the navigation system [32], which helped with a U-shaped registration mold consisting of special fiducial markers [32].

The surgery included a pinpoint access cut by a surgical drill [32].

#### 3.3.9. Sequestrectomy at Stage 2 of Medication-Related Osteonecrosis of the Jaws

One study [16] above the twenty-nine studies included [14,15,16,17,18,19,20,21,22,23,24,25,26,27,28,29,30,31,32,33,34,35,36,37,38,39,40,41,42] described the sequestrectomy at stage 2 of the medication-related osteonecrosis of jaw (MRONJ) procedure on 86-year-old partially edentulous female patient (0.47%) under pharmacological treatment with aledronic acid for 5 years and denosumab once 3 months before the surgery [16].

The initial radiographic evaluations were performed using OPT and CBCT [16].

The MRONJ extended from the inferior right canine to the inferior left second premolar, exposing bone for ~2 × 1.5 cm. The lesion extended over the symphysis close to the left mental foramen, and the mandible’s inferior border residual height was ~7 mm [16].

The Dynamic Computer Navigation devices used were Navident version R2.1.1.^®^ as navigation software and Navident^®^ as the navigation system, which helped with the points of the right mandibular canine, the bilateral mental nerve, and a temporary screw [16].

The surgery included sequestrectomy and saucerization of the infected bony margin [16].

The follow-up was positive with a lip dysesthesia resolution at 1 month [16].

The clinician reported that the Dynamic Computer Navigation devices overcame the issue of the mobility of the mandible [16].

#### 3.3.10. Osteoplasty Before the Implant Placement

One study [21] above the 29 included [14,15,16,17,18,19,20,21,22,23,24,25,26,27,28,29,30,31,32,33,34,35,36,37,38,39,40,41,42], described osteoplasty before the implant placement in a 44-year-old partially edentulous female patient (0.47%) with severe generalized periodontitis [21].

The study carried out a dental impression technique using a digital intraoral scanner [21].

The initial radiographic evaluations were performed using CBCT [21].

The pneumatization of the maxillary sinuses and severe bone resorption were present in the posterior maxilla [21].

The Dynamic Computer Navigation devices used were iPLAN Navigator BrainLAB^®^ as planning software, BrainLAB^®^ as navigation software, and VectorVision2^®^ as navigation system, which helped with parallel pins [21].

The surgery included the osteotomy of the maxilla [21]. Data on surgical duration are not available.

One study reported a mean deviation of 1.3 ± 0.39 mm (from 0.8 to 1.7 mm) of the Dynamic Computer Navigation devices [21].

### 3.4. Bone Augmentation Surgeries

#### 3.4.1. Sinus Elevation Surgeries

Three studies [19,23,39] of the twenty-nine studies included [14,15,16,17,18,19,20,21,22,23,24,25,26,27,28,29,30,31,32,33,34,35,36,37,38,39,40,41,42] described surgeries of sinus elevation and incorporated a total of 64 participants (29.91%), 30 males and 32 females between 27 and 78 years old, with a mean age of 49.47 [19,23,39].

One study reported the surgery of the sinus floor elevation in a 27-year-old woman (1.56%) through a lateral access [19], while another study reported the surgery of the sinus floor elevation in 28 patients (43.75%) through a transcrestal access [39], and another study reported the surgery of the sinus floor elevation in 35 patients (54.68%) through both accesses based on the residual alveolar bone height [23].

Two studies carried out conventional dental impressions with silicone elastomer [39] and a U-shaped tube placed with polyether impression material (3M ESPE) [23].

The initial radiographic evaluation was performed using CBCT [19,23,39], aided by the positioning of the head tracker and a trace registration device [19].

In the 27-year-old female, the thickness of the residual bone in the upper first molar region was 6 mm [19]; in 28 patients, the thickness was at the bottom of the maxillary sinus floor [39]; and in 35 patients, the thickness of the residual bone in the maxillary region was classified as 3 mm ≤ RBH < 6 mm and 6 mm ≤ RBH < 10 mm [23].

The Dynamic Computer Navigation devices used were Dcarer^®^ as planning and navigation software [23,39] and as a navigation system [23]. Navident version 3.0^®^ as a navigation software and Navident unit^®^ as a navigation system [19] helped with the U-shaped tube [23].

The surgery included the setup of the lateral bone window with an angle deviation of 8.93°, an entry deviation of 2.83 mm, a depth deviation of 0.29 mm, and a linear lateral deviation of 2.52 mm [19], and the drilling and the osteotomy accomplished the transcrestal sinus floor elevation [39].

#### 3.4.2. “Sandwich” Procedure

One study [26] of the twenty-nine studies included [14,15,16,17,18,19,20,21,22,23,24,25,26,27,28,29,30,31,32,33,34,35,36,37,38,39,40,41,42] described the “sandwich” surgery procedure on a 56-year-old female patient (0.47%).

The study carried out a dental impression technique using a digital intraoral scanner [26].

The initial radiographic evaluations were performed using CBCT aided by landmarks acquired by the digital intraoral scanner [26].

The Dynamic Computer Navigation devices used were not defined but were helped by tracer tip verification [26].

The surgery included a horizontal osteotomy located at the posterior mandible region with a depth of 2 mm above the mandibular canal and a linear lateral incision of 2 mm, distal to the last residual tooth [26].

The follow-up was performed at two weeks and one month [26].

#### 3.4.3. Autogenous Bone Ring Technique

One study [22] of the twenty studies included [14,15,16,17,18,19,20,21,22,23,24,25,26,27,28,29,30,31,32,33,34,35,36,37,38,39,40,41,42] described the autogenous bone ring technique surgery procedure [22]. There are no available data for the sample size.

The study carried out a dental impression technique using a digital intraoral scanner [22].

The initial radiographic evaluations were performed using CBCT [22].

The Dynamic Computer Navigation devices used were Mimics Medical 20.0^®^, which helped with the corresponding anatomical features of the teeth [22].

The surgery included making an incision at the crest of the ridge, preparing the bone ring bed, and embedding the bone ring in the mandibular branch [22].

### 3.5. Quality Assessment

The risk of bias and the related quality assessment of the case report (Table S2), case series (Table S3), RCT (Table S4), and prospective study (Table S5) included in the present systematic review are reported in Supplementary File S2.

The inter-rater reliability with Cohen’s kappa was 0.82 for the risk-of-bias assessment.

## 4. Discussion

Among the 29 studies included [14,15,16,17,18,19,20,21,22,23,24,25,26,27,28,29,30,31,32,33,34,35,36,37,38,39,40,41,42] aiming to identify the reliability, accuracy, patient- and clinician-reported usability, acceptability, and satisfaction of dynamic computer-assisted navigation systems in dentoalveolar and maxillary bone augmentation surgeries, the majority (*n* = 20) were case reports [15,16,17,19,21,22,24,26,27,28,29,30,31,32,33,35,36,37,40,41], reflecting the fragmented and preliminary nature of the current evidence. Indeed, although dynamic navigation was introduced in neurosurgery through Roberts’ pioneering work in the 1980s, its integration into oral and maxillofacial surgery began in 1995, with widespread adoption over the past three decades, especially in dental implantology [43].

However, as evidenced by this review, its application in dentoalveolar surgery is a relatively recent development. Except for a case report from 2006 [32], all included studies on dentoalveolar procedures were published between 2014 and 2024 [14,15,16,17,18,19,20,21,22,23,24,25,26,27,28,29,30,31,33,34,35,36,37,38,39,40,41,42]. Its use in maxillary bone augmentation is even more recent, with all relevant studies published between 2021 and 2024 [19,22,23,26,39]. Consequently, dentoalveolar procedures accounted for the majority of reported cases (69.62%), while maxillary bone augmentation represented a smaller proportion (30.38%) of dynamic navigation system applications.

### 4.1. Dentoalveolar Surgeries

#### 4.1.1. Complex Tooth Extractions

Two studies [30,33] reported the use of dynamic computer-assisted navigation systems in complex tooth extractions. One involved the removal of displaced molar roots in direct contact with the IAN [33], and the other two molars were traumatically displaced into the maxillary sinus, complicated by gingival laceration, an orifice fistula, an alveolar fracture, and bilateral condylar fractures resulting from prior treatment [30]. These cases underscore the dynamic navigation system’s potential in managing surgically demanding scenarios, particularly those involving limited access or proximity to critical anatomical structures. By enabling detailed preoperative planning and real-time, three-dimensional intraoperative guidance, the technology enhances anatomical orientation and may mitigate the risk of complications [8,43]. Notably, in the study by Wang et al. [30], dynamic navigation also facilitated intraoperative assessment of mandibular and condylar fracture reduction through the superimposition of preoperative plans and real-time imaging.

No complications were reported in either study, including at the 6-month follow-up [30,33], suggesting a favorable safety profile even in high-risk cases.

The mean surgical duration was approximately 50 min, with registration taking an additional 5 min [30,33]. Although slightly longer than standard extraction times, the added precision and complication-free outcomes suggest that dynamic navigation systems may offer significant clinical value in complex dental extractions.

#### 4.1.2. Supernumerary Tooth Extractions

Five studies [17,20,35,37,42] described the supernumerary tooth extraction in 21/214 subjects, as a part of a multidisciplinary approach to dental anomalies to achieve proper function, shape, or aesthetics [44]. The prevalence of supernumerary teeth, defined as additional teeth to the normal series that can be spotted in any dental arch region [45], is estimated between 0.2 and 3% and is more common in males [46] (M:F = 3.1:1.7 [47]), in accordance with the present results (M:F = 6:1). Moreover, the low mean patient age of 10.09 years (range: 7–29) aligns with current recommendations advocating the early extraction of both unerupted and erupted supernumerary teeth upon diagnosis [48]. Early intervention helps prevent secondary complications such as altered eruption patterns, displacement, and root hypoplasia of adjacent permanent teeth [45], as observed in one case in the present review [42].

Four studies [20,35,37,42] reported the cases of 19 patients with mesiodens, a maxillary supernumerary tooth in the palatal region between central incisors. The maxilla is the most frequent region in which the supernumerary teeth were found (80–90% of cases) [49], and mesiodens are the most common [50], as manifested in 0.15–1.9% subjects [49]. Patients with two supernumerary teeth were described in two studies [35,37], which are rare, representing less than 1% cases according to the literature [47]. Finally, two studies [17,20] described cases of mandibular supernumerary teeth in the second premolar [17] and first molar regions [17,20], defined as paramolars and distomolars [50], which have a higher prevalence in the mandible compared to other supernumerary tooth types [51].

Considering that the extractions of the supernumerary teeth included in the present systematic review had anatomical challenges, such as the proximity to the nasal cavity and maxillary sinus [35], to the roots of the adjacent tooth [17,37] or mental foramen [20], the use of dynamic computer-assisted navigation systems in supernumerary tooth extraction appears to be advantageous, even taking into account that in pediatric patients, more severe non-cooperative behavior was recorded during tooth extraction procedures compared to non-extractive previous dental appointments [52]. This technology enabled the precise localization of the supernumerary teeth, minimizing intraoperative duration and complications, particularly in surgeries with anatomical challenges [43]. In fact, the study by Wang et al. [42] showed statistically significantly higher accuracy comparing the extractions of supernumerary teeth performed with dynamic computer-assisted navigation systems vs. freehand surgeries, evaluating in the entry deviation (all the teeth were exposed at the planned access point in the navigation group), depth deviation (extra bone removed in length), and linear lateral deviation (extra bone removed in width).

Notably, one patient who underwent supernumerary tooth extraction with this technology reported only mild pain, resolved within 2 weeks, and experienced no significant discomfort, suggesting a positive surgical experience [17]. However, Wang et al. [42], who compared the supernumerary tooth extraction performed using dynamic computer-assisted navigation systems vs. freehand, did not find a statistical difference in the patient’s reported pain on the second day after surgery. The mean values of the Visual Analogue Scale were 1.37 ± 0.16 and 1.23 ± 0.17, respectively [42]. So the dynamic computer-assisted navigation systems did not differ in terms of patient-reported pain from freehand.

Three studies [17,37,42] described the duration of surgery of supernumerary tooth extraction with a mean duration of 18 min. Comparing the operation time between navigation surgeries vs. freehand surgery, Wang et al. [42] registered a shorter duration in the navigation group, while the pre-operative planning times were longer.

#### 4.1.3. Lower Third Molars Extractions

High-risk extractions of lower third molars in proximity to the IAN [18,25], sublingual [15,38], pterygomandibular, and lateral pharyngeal space [38] performed with dynamic computer-assisted navigation systems were described in four studies [15,18,25,38], involving 96 patients (45 males, 51 females, from 18 to 56 years, with a mean age of 24.34).

Notably, no complications were reported in the extraction of 80 deeply impacted horizontal lower third molars performed using dynamic computer-assisted navigation, in contrast to 80 cases managed freehand, which resulted in second molar damage in three cases (3.75%) and lip dysesthesia in four cases (5%) [18]. The latter finding, indicative of IAN injury, aligns with reported prevalence rates of 8–10% in high-risk surgeries, compared to 0.4–0.8% in routine third molar extractions [53,54].

Similarly, no cases of lingual nerve injury were reported. This contrasts with published rates of permanent lingual nerve injury ranging from 0.3 to 0.6% and transient injury rates as high as 15% following lower third molar extraction [55]. Notably, several surgeries involved displaced teeth located in confined surgical fields where hemorrhage could impair visibility, and blind probing might result in further displacement [56]. The absence of IAN or lingual nerve injury in such high-risk scenarios suggests that dynamic computer-assisted navigation systems may significantly reduce iatrogenic complications, representing a valuable tool to improve lower third molar surgery safety.

Regarding operative duration, the reported extraction times for third molars using dynamic computer-assisted navigation ranged from 15 [38] to about 37 min [18] (the latter related to horizontal lower third molars), with a mean duration of 25.5 min. In particular, FangFang et al. [18] found that the horizontally impacted lower third molar extraction required 37 ± 5 min with dynamic navigation, comparable to 36 ± 3 min using the freehand technique. Although the total surgical time differed only minimally (1 ± 2 min), dynamic navigation significantly reduced the intraoperative phase to 22 ± 3 min, with about 15 min required for preoperative planning and system setup, representing about 40% of the total procedure time [18]. Similarly, Pillai et al. [57] reported a mean total procedure time of 40.4 min for dynamic navigation, slightly exceeding the range observed in the present review but still shorter than laser-assisted (42.8 min) and freehand (45.2 min) techniques and comparable to piezoelectric surgery (38.6 min).

These findings suggest that while dynamic navigation may require additional preoperative preparation, it offers notable reductions in intraoperative time. This efficiency could translate into clinical advantages, as shorter surgery durations of third molar extractions have been associated with improved patient satisfaction [58]. However, no patient-reported outcome measures were included in the studies analyzed in this systematic review, highlighting the need for future research on patient-centered benefits.

#### 4.1.4. Coronectomy of Lower Third Molars in Close Proximity to the IAN

One study [14] evaluated the use of dynamic computer-assisted navigation systems for performing coronectomy in 12 patients (M:F = 5:7; mean age: 28.67 years; age range: 24–32). Coronectomy, introduced by Knutsson et al. [59], is a nerve-sparing technique that is still debated and is particularly indicated for patients over the age of 25 [54], involving the removal of the tooth crown while retaining the roots within the socket to prevent nerve injury. Dynamic navigation was specifically employed to enhance the coronectomy precision, as maintaining a residual root length of less than 7.6 mm and positioning it at least 5 mm below the bone margin is critical to reducing the root exposure risk [60]. The procedure demonstrated favorable accuracy, with a reported root mean square deviation of 0.69 ± 0.21 mm, although maximum deviations ranged from 1.45 ± 0.83 mm to 1.87 ± 0.63 mm [14].

Analyzing this data, it should be considered that the clinician-reported usability was limited to the difficulties found during surgery, because dynamic computer-assisted navigation systems are not equipped for and lack flexibility to handle irregular boundaries for the tooth sectioning [14], which could compromise the accuracy, also increasing the operative time. A dedicated handpiece could improve the accuracy and duration time (30–40 min reported) of coronectomies through dynamic navigation systems.

Notably, no postoperative complications were observed. At 3 months, no cases of infection, pulpitis, dry socket, nerve injury, or root migration were reported, supporting the safety of dynamic computer-assisted navigation systems for coronectomy [14].

#### 4.1.5. Foreign Bodies, Bone Graft Fixing Screws, and Dental Implants Removal

Six studies [24,27,28,29,31,41] described the removal of a foreign body (six patients), two studies [20,34] dental implants (seven patients) and one study [32] bone graft fixing screws (one patient), using dynamic computer-assisted navigation systems.

In 80% of cases, the foreign bodies were located for an idiopathic reason, in complex operative-access areas, such as posterior mandible [14] and the lower first premolar region [15] in proximity to the IAN [14,15]; mandibular lingual soft tissue region [11]; medial pterygoid muscle [18]; and pterygomandibular space, medial to the mandibular ramus [16].

This would have made the freehand retrieval and removal of the foreign bodies more difficult. According to the literature, dynamic computer-assisted navigation systems were recommended for challenging cases that may cause complications, such as displaced foreign bodies, particularly if there are multiple [43]. In these cases, the major advantage offered by the use of technology could be the recognition of the position of the foreign body, fixing screws, and displaced implants, allowing the surgeon to also recognize tiny objects such as broken dental needles, improving the accuracy [9] and reducing the surgery risk and duration [29,31].

Surgery duration had a mean time of 31.7 min [24,27,29] without negative feedback reported from the clinician or patient.

#### 4.1.6. Osteolytic Lesions of the Jaws

Three studies [16,36,40] reported osteolytic lesions of the jaws treated using the dynamic computer-assisted navigation systems; in particular, two studies [36,40] described cyst removal surgery (one in the mandibular and one in the maxillary molar region), and one study [16] performed the sequestrectomy to treat stage 2 of MRONJ.

The treatment of maxillofacial osteolytic lesions requires detailed anatomical knowledge and related three-dimensional surgical planning, particularly regarding resection margins [43]. Dynamic computer-assisted navigation systems serve as a valuable adjunct in identifying anatomical structures and lesion extension [43]. This technology facilitates cyst enucleation and removal while preserving bone during osteotomies [43].

In the MRONJ case, the osteolytic and sclerotic lesions were in the symphyseal region, adjacent to the mental foramen [16]. Due to the case complexity, sequestrectomy and saucerization were performed using dynamic computer-assisted navigation to minimize the IAN injury risk and to ensure the complete removal of the affected bone [61]. The procedure was completed without complications, and the patient achieved full recovery at 1 month, confirming the safety and accuracy of this technology [16].

While no patient outcome was reported, clinicians stated that the devices overcame mandible mobility issues during sequestrectomy, resulting in a better performance.

#### 4.1.7. Osteoplasty Prior to Implant Placement

One included study [21] reported a case of osteoplasty before the implant placement with the use of dynamic computer-assisted navigation systems [21]. After the extractions of the remaining teeth in the anterior maxillary region [21], the technology was employed to perform real-time osteoplasty before the implant placement [21].

Previous studies demonstrated that dynamic computer-assisted navigation systems had higher accuracy in guiding osteotomy [9]. Dianat et al. [62] compared the osteotomy accuracy before root-end resection performed through dynamic computer-assisted navigation systems vs. freehand on human cadavers, reporting significantly greater accuracy using technology, in particular, angular and linear accuracy. In addition, shorter surgical time was registered [62]. Similarly, osteoplasty before implant placement showed promising results, with a mean deviation of 1.3 ± 0.39 mm (from 0.8 to 1.7 mm) [21].

### 4.2. Bone Augmentation

#### 4.2.1. Maxillary Sinus Floor Elevation

Maxillary sinus floor elevation (MSFE) was performed in three studies [19,23,39] through dynamic computer-assisted navigation systems on 64 patients (M:F = 32:32) with a mean age of 47.83 (range: 27–78), but its accuracy was assessed in only one case [19] of maxillary sinus lateral access and subsequent navigated implant placement. Deviation data reported from the lateral access performed with dynamic computer-assisted navigation systems were angle deviation 8.93°, entry deviation 2.83 mm, depth deviation 0.29 mm, and linear lateral deviation 2.52 mm, while those from implant placement were entry deviation 0.03 mm, apex deviation 0.82 mm, vertical apex deviation 0.82 mm, and angle deviation 0° [19]. Notably, these findings indicate lower dynamic computer-assisted navigation system performance in MSFE compared to dental implant placement, despite both being performed by the same surgeon. However, even in this case, the angular (3.68°) and entry point (1.03 mm) deviations recorded for implant placement were lower than the averages reported in the literature for dynamic navigation [4]. The lower accuracy in MSFE may be attributed to clinician-reported challenges in identifying soft tissues during surgery, particularly the sinus membrane [19]. The use of dynamic computer-assisted navigation in this context requires enhanced tactile sensitivity, as the surgeon must distinguish between soft and hard tissues while operating with limited direct visual orientation, relying instead on a computer interface linked to a navigation-enabled handpiece. This highlights one of the major technical limitations of dynamic navigation systems: the limited ability to adequately differentiate soft tissue boundaries intraoperatively. Since sinus membrane perforation is a primary complication in MSFE, the suboptimal ability to distinguish the membrane from surrounding tissues may fall short in ensuring surgical safety.

Another included study reported navigated MSFE via either a lateral or a transcrestal approach based on residual bone height in 35 patients; notably, no complications were observed [23]. Given that Schneiderian membrane perforation is the most common MSFE-related complication, with an estimated incidence of 20–44% [63], the absence of such events in this study may suggest a favorable safety profile of dynamic navigation in MSFE.

While no patient-reported outcomes were documented for dynamic navigation in this review, existing data on freehand lateral MSFE indicate moderate postoperative pain levels, typically diminishing within two days [63].

The use of dynamic computer-assisted navigation systems in MSFE surgery may demonstrate promising potential, particularly in terms of safety, as no complications were reported in the included studies. However, difficulties in accurately identifying the sinus membrane may compromise both the precision and safety of the procedure. Further studies with larger sample sizes are warranted to more thoroughly evaluate the accuracy and clinical reliability of this technology in MSFE intervention procedures.

#### 4.2.2. Other Vertical Bone Augmentation Procedures

Two studies [22,26] reported the use of dynamic navigation systems in vertical bone augmentation, specifically the “Sandwich” osteotomy [26] and a modified bone ring technique [22], and both advocated for improving surgical precision, particularly in relation to the IAN. “Sandwich” osteotomy involves horizontal and vertical mandibular osteotomies to create space for bone grafting, thereby increasing bone height and width in atrophic posterior mandibles with limited intermaxillary space and insufficient bone volume above the IAN [64]. The bone ring technique, by contrast, is a one-stage procedure designed to overcome limitations of delayed implant placement following only grafting. It includes harvesting an autogenous bone ring, placing it in the edentulous site, filling residual gaps with graft material, and simultaneously placing the implant into the ring [22,65].

Dynamic computer-assisted navigation systems demonstrated a clear safety advantage in both reported cases of vertical bone augmentation [22,26], with no complications observed. The technology provided real-time visualization of surgical instruments relative to critical anatomical structures, including the IAN, thereby enhancing the precision of osteotomies and minimizing the risk of iatrogenic injury [26]. This contrasts sharply with existing literature on freehand “Sandwich” osteotomies, where the incidence of IAN paresthesia ranges from 5.6 to 100%, highlighting it as one of the most frequent and significant complications [66]. In the navigated “Sandwich” procedure [26], no neurosensory disturbances were recorded. This favorable outcome may also be attributed to the use of a piezoelectric handpiece, which is associated with a reduced risk of nerve injury [67]. Although bone resorption data were not reported, and follow-up was limited to 2 weeks and 1 month [26], and these short-term outcomes are consistent with the expected postoperative course. The literature suggests a typical bone healing period of 3–6 months, during which some degree of resorption is anticipated following augmentation procedures [66].

While the use of dynamic navigation in vertical bone augmentation remains scarcely documented, the absence of complications and favorable early outcomes [22,26] highlights its potential value. Further studies with larger cohorts are warranted to develop standardized protocols in complex augmentation procedures.

### 4.3. Clinical Relevance

The findings of this systematic review indicate that dynamic computer-assisted navigation systems demonstrate higher reliability in dentoalveolar surgery compared to maxillary bone augmentation procedures and safety in both, even in complex clinical scenarios involving high anatomical risk.

Despite these promising findings, the dynamic computer-assisted navigation system is not without drawbacks. Limitations in soft tissue recognition and instrument incompatibility can compromise its accuracy in certain surgical cases. Moreover, the high technology initial cost, the time needed for the setup, and the required operator training may restrict its widespread adoption. These limitations should be carefully weighed when considering the integration of navigation systems into surgical practice in a dental setting.

#### 4.3.1. Accuracy, Surgical Performance, and Duration

In dentoalveolar surgeries, dynamic computer-assisted navigation systems demonstrated accuracy comparable to or exceeding that of freehand techniques, particularly in the extraction of supernumerary teeth. This was especially evident in pediatric cases, where the technology enabled the precise localization of impacted teeth, minimized intraoperative time, and reduced surgical trauma in anatomically challenging situations. These benefits are particularly important given the increased incidence of non-cooperative behavior in children during extraction procedures.

In complex extractions—such as those involving displaced roots or foreign bodies near critical anatomical structures—dynamic navigation provided precise, real-time three-dimensional guidance and improved spatial orientation. In certain cases, the technology also enabled the intraoperative verification of mandibular and condylar fracture reduction, further illustrating its value in high-complexity surgical contexts.

In third molar extractions, especially those with deep impactions or proximity to the inferior alveolar or lingual nerve, dynamic navigation enhanced surgical control and reduced both angular and entry point deviation. These improvements contributed to shorter intraoperative times, despite a minor increase in total procedural time due to preoperative calibration. The increased accuracy and reduced surgical time may have played a role in the absence of complications, and support improved patient compliance and satisfaction.

In coronectomy procedures, although the overall accuracy was acceptable, some variability in deviation was observed, highlighting limitations related to irregular sectioning boundaries and the absence of navigation-compatible handpieces specifically designed for crown sectioning. Nonetheless, these procedures were completed safely, with no complications reported at follow-up.

In contrast, lower accuracy was noted in MSFE procedures compared to implant placement, even when conducted by the same surgeon. This was primarily attributed to the difficulty in differentiating soft tissue structures, such as the Schneiderian membrane, from surrounding tissues. This challenge is compounded by reduced tactile feedback and reliance on a computer interface for visual guidance. Despite these limitations, no complications were reported in either lateral or transcrestal MSFE approaches, suggesting that the technology still provides a safe and controlled surgical environment.

Vertical bone augmentation procedures, including the “Sandwich” osteotomy and the autogenous bone ring technique, also benefited from the dynamic navigation-enhanced accuracy. No complications were recorded, even in cases close to the IAN. This contrasts sharply with higher complication rates documented for similar procedures performed freehand, underscoring the role of navigation in preserving anatomical integrity.

While the integration of dynamic navigation systems resulted in a slight increase in total procedure time, mainly due to preoperative calibration, the operative phase was generally shortened, particularly in third molar extractions. These reductions were not only operationally advantageous but may also translate into improved intraoperative efficiency. No specific data were available regarding surgical time in bone augmentation procedures.

Collectively, these findings highlight the broad applicability and clinical potential of dynamic computer-assisted navigation systems in enhancing surgical accuracy across a range of oral and maxillofacial procedures. While some limitations remain—particularly in soft tissue identification and instrument compatibility—the technology consistently improves anatomical precision, intraoperative control, and overall surgical safety.

#### 4.3.2. Complication Rates, Safety, and Patient- and Clinician-Reported Outcomes

The evidence from this systematic review highlights a consistently favorable safety profile of dynamic computer-assisted navigation systems across various surgeries. Notably, no intraoperative or postoperative complications were reported in any of the included studies, even in high-risk cases. Despite technical limitations, complication rates were consistently lower than the average reported for freehand surgeries. This trend was observed across all surgical categories reviewed, highlighting the potential of dynamic navigation to enhance safety through improved anatomical control and precision, reducing surgical risk, even in complex procedures involving critical anatomical structures [68].

In third molar extractions, particularly those involving proximity to the IAN, the dynamic navigation was associated with no complications, contrasting with the freehand approach, which showed measurable rates of nerve injury and adjacent tooth damage. Similarly, coronectomy procedures and supernumerary tooth extractions were completed without adverse events, even in anatomically challenging contexts.

In MSFE procedures, where Schneiderian membrane perforation is common in freehand surgery (20–44%), dynamic navigation also resulted in no reported complications, despite some limitations in soft tissue discrimination [19]. Additionally, in vertical bone augmentation procedures such as the “Sandwich” osteotomy, dynamic navigation prevented nerve injury, which is a frequent issue in freehand techniques.

Patient-reported outcomes were scarcely addressed. Limited available data, such as those related to supernumerary tooth extractions, revealed no significant differences in perceived postoperative pain between dynamic navigation and freehand techniques.

In contrast, clinician-reported outcomes offered more insight. Surgeons noted improved intraoperative control, including reduced mandibular mobility, which facilitated surgical execution. However, the primary limitation remained the difficulty in differentiating between soft and hard tissues—a factor particularly critical in MSFE procedures where accurate identification of the sinus membrane is essential.

### 4.4. Strengths, Limitations, and Future Directions

The present system review represents the first attempt to analyze and synthesize the reliability, accuracy, and usability of dynamic computer-assisted navigation system devices in dentoalveolar and bone augmentation surgeries. Furthermore, a key strength lies in the inclusion of recent studies, which provide up-to-date insights into oral surgery.

However, study limitations may be taken into account, such as the heterogeneity among the included studies concerning the type of surgery and the investigated outcomes, which reduced the overall generalizability of the findings, as well as the possibility to conduct a quantitative results analysis through meta-analysis.

In addition, only a few studies included addressed both the primary and secondary outcomes investigated in the present review, limiting the possibility of achieving comprehensive knowledge on reliability, accuracy, and usability of the dynamic computer-assisted navigation system for all dentoalveolar surgeries and bone augmentation surgeries.

Secondly, a significant constraint lies in the fragmented nature of the evidence, predominantly composed of case reports. While these provide valuable preliminary insights, their inherent design limitations, such as small sample sizes and lack of control groups, restrict the ability to draw definitive conclusions. To address these gaps, future research should prioritize high-quality comparative studies, including RCTs where feasible. These studies should focus on long-term outcomes, complication rates, and cost-effectiveness, providing more insight into clinical potential advantages and applications of dynamic computer-assisted navigation systems in dentoalveolar and bone augmentation surgeries.

Hence, further advances in the use of this technology should be directed to standardize protocols for dynamic computer-assisted navigation systems; improve technology to overcome the issue of navigation systems in differentiating soft tissue and hard tissue; and develop new dedicated handpieces (such as to facilitate the dental crown sectioning during coronectomy) for dentoalveolar surgeries and bone augmentation surgeries.

Beyond technological and research advancements, the integration of dynamic navigation systems into early training curricula should be highly recommended. Introducing these technologies at an undergraduate or postgraduate level can significantly reduce the learning curve for future clinicians. This approach should also consider strategies to make these advanced technologies more affordable, thereby promoting their widespread accessibility and integration into the dental setting.

## 5. Conclusions

The present systematic review, including 29 studies and 214 patients, evaluated the reliability, accuracy, patient- and clinician-reported usability, acceptability, and satisfaction of dynamic computer-assisted navigation systems in dentoalveolar surgery and maxillary bone augmentation.

Dynamic navigation systems demonstrated high reliability and accuracy in both dentoalveolar surgery and maxillary bone augmentation, with a notably lower incidence of complications compared to freehand techniques, even in complex cases. While the initial setup increased overall operation time slightly, actual surgical time was often reduced, potentially improving patient satisfaction, an outcome that unfortunately has not been extensively investigated.

Despite these advantages, technological limitations exist, particularly in navigating soft tissues during sinus lift procedures, high initial and maintenance costs, and the necessary learning curve.

Considering the limited and fragmented current knowledge, in fact, most of the included studies were case reports, and future research should focus on refining the technology to better differentiate tissue types and enhance accuracy in procedures like sinus lifts and investigate patient-reported outcomes, particularly satisfaction, to guide targeted improvements.

## Figures and Tables

**Table 1:** Extraction data table of the included studies, populations, and intervention characteristics: study characteristics (first author; publication years; publication journal; reference; design; quality judgment; source of funding); population characteristics (ample size, gender ratio, mean age and/or range, comorbidities, ongoing pharmacological treatment, dentition status); oral surgery characteristics (type, surgery steps performed with dynamic computer-assisted navigation, location, other characteristics); type of intervention (radiographic imaging, guidance method for imaging, planning software, navigation software, navigation system, guidance methods for navigation, dental impression technique).

Studies	Population	Oral Surgery Characteristics	Type of Intervention
Casap N., 2006 [32] Int J Oral Maxillofac Implants Case report Included No Funding	Sample size: n.1 Gender ratio: 1F Mean/Range age: 24 y.o. Comorbidities: MD Ongoing pharmacological treatment: MD Dentition status: partially dentulous	Type: Removal of bone graft fixing screws Surgery step(s) performed with DCAN: pinpoint access cut by a surgical drill Location: maxillary left first molar and between the first and second premolar regions Other characteristics: None	Radiographic imaging: CBCT Guidance method for imaging: U-shaped registration mold consisting of special fiducial markers Planning software: IGI software^®^ Navigation software: IGI software^®^ Navigation system: specialized trackable handpiece of the IGI system Guidance method for navigation: U-shaped registration mold consisting of special fiducial markers Dental impression technique: MD
Chen K.J., 2020 [31] ScienceDirect Case report Included No Funding	Sample size: n.1 Gender ratio: 1F Mean/Range age: 36 y.o. Comorbidities: MD Ongoing pharmacological treatment: MD Dentition status: dentulous	Type: Removal of foreign body (broken dental needle) Surgery step(s) performed with DCAN: retrieval of the needle Location: in the medial pterygoid muscle Other characteristics: MD	Radiographic imaging: CBCT Guidance method for imaging: vinylpolysiloxane customized bite block Planning software: MD Navigation software: MD Navigation system: StealthStation^TM^ S8 (Medtronic)^®^ Guidance method for navigation: MD Dental impression technique: MD
Chen S., 2019 [24] Medicine Case report Included No Funding	Sample size: n.1 Gender ratio: 1M Mean/Range age: 41y.o. Comorbidities: MD Ongoing pharmacological treatment: MD Dentition status: MD	Type: Removal of foreign body (high-speed fissure bur) Surgery step(s) performed with DCAN: detection of the foreign metallic body Location: mandibular lingual soft tissue region Other characteristics: limited mouth opening (2cm), numbness on the left side of the lower lip	Radiographic imaging: OPT, CBCT Guidance method for imaging: MD Planning software: AccuNavi-A^®^ Navigation software: MD Navigation system: AccuNavi-A^®^ Guidance method for navigation: customized mandible reference frame Dental impression technique: MD
Chen Y.T., 2020 [16] Oral Maxillofac Surg Case report Included No Funding	Sample size: n.1 Gender ratio: 1F Mean/Range age: 86 y.o. Comorbidities: N/D Ongoing pharmacological treatment: aledronic acid for 5 years and denosumab once 3 months before Dentition status: partially edentulous	Type: Sequestrectomy (stage 2 MRONJ) Surgery step(s) performed with DCAN: sequestrectomy and saucerization of infected bony margin, mental and IAN preservation, and incisive branch anatomy Location: from the inferior right canine to the inferior left second premolar region Other characteristics: exposed bone for ~2 × 1.5 cm, osteolytic and sclerotic lesion over the symphysis close to the left mental foramen and a residual inferior border of the mandible ~7 mm in height	Radiographic imaging: OPT, CBCT Guidance method for imaging: MD Planning software: MD Navigation software: Navident version R2.1.1. ^®^ Navigation system: Navident^®^ Guidance method for navigation: Right mandibular canine, bilateral mental nerve, and a temporary screw Dental impression technique: MD
Dotia A., 2024 [19] Cureus Case report Included No Funding	Sample size: n.1 Gender ratio: 1F Mean/Range age: 27 y.o. Comorbidities: MD Ongoing pharmacological treatment: MD Dentition status: N/D	Type: SFE procedure Surgery step(s) performed with DCAN: lateral window surgery Location: upper first molar region Other characteristics: residual bone height available was 6 mm	Radiographic imaging: CBCT Guidance method for imaging: positioning of the head tracker and a trace registration device Planning software: MD Navigation software: Navident version 3.0^®^ Navigation system: Navident unit^®^ Guidance method for navigation: MD Dental impression technique: MD
FangFang X., 2024 [18] BMC Oral Health RCT Low Risk Shaanxi Provincial Health and Medical Research and Innovation Capacity Improvement Plan Project; Clinical Research Project of Xi’an Jiaotong University Stomatological Hospital	Sample size: n.80 Gender ratio: 35M/45F Mean/Range age: 23.7 y.o./18–35 y.o. Comorbidities: None Ongoing pharmacological treatment: MD Dentition status: dentulous	Type: Third molar extraction (Deep horizontal) Surgery step(s) performed with DCAN: ostectomy of the bone covering the crown, odontotomy, and tooth extraction Location: mandibular third molar region Other characteristics: proximity to the IAN, adjacent second molar	Radiographic imaging: CBCT Guidance method for imaging: U-shaped tube filled with silicone rubber Planning software: Mimics 21.0^®^ Navigation software: MD Navigation system: MD Guidance method for navigation: silicone rubber model fixed with a dental resin in the contralateral area of the mandible Dental impression technique: MD
FangFang X., 2024 [17] Am J Case Rep Case report Included Shaanxi Provincial Health and Medical Research and Innovation Capacity Improvement Plan Project, Clinical Research Project of Xi’an Jiaotong University Stomatological Hospital	Sample size: n.1 Gender ratio: 1F Mean/Range age: 22 y.o. Comorbidities: MD Ongoing pharmacological treatment: MD Dentition status: dentulous	Type: Extraction of supernumerary teeth Surgery step(s) performed with DCAN: osteotomy Location: left lingual side between the lower second premolar and first molar Other characteristics: proximity to the roots of the lower second premolar and first molar	Radiographic imaging: CBCT Guidance method for imaging: radiopaque spatial markers Planning software: Dcarer^®^ Navigation software: Dcarer^®^ Navigation system: DHC-DI2^®^ Guidance method for navigation: emitting infrared light to the detection camera on spatial markers Dental impression technique: N/D
Felice P., 2021 [26] Methods Protoc Case report Included No Funding	Sample size: n.1 Gender ratio: 1F Mean/Range age: 56 y.o. Comorbidities: None Ongoing pharmacological treatment: N/D Dentition status: N/D	Type: “Sandwich” Technique Surgery step(s) performed with DCAN: horizontal osteotomy Location: posterior mandible region Other characteristics: 2 mm above the mandibular canal; 2 mm distal to the last residual tooth	Radiographic imaging: CBCT Guidance method for imaging: landmarks acquired by the digital intraoral scan Planning software: MD Navigation software: MD Navigation system: MD Guidance method for navigation: tracer tip verification Dental impression technique: digital impression
Guo Y., 2015 [38] J Oral Maxillofac Surg Case series Included China Scholarship Council	Sample size: n. 12 Gender ratio: 7 M/5F Mean/Range age: 24.23 y.o./18–42 y.o. Comorbidities: MD Ongoing pharmacological treatment: MD Dentition status: dentulous	Type: Third molar extraction Surgery step(s) performed with DCAN: clamping and retrieving of the molars Location: Sublingual space, *n* = 7; Pterygo-mandibular space, *n* = 4; lateral pharyngeal space, *n* = 1 Other characteristics: swelling, trismus, swallowing pain	Radiographic imaging: CBCT Guidance method for imaging: MD Planning software: MD Navigation software: MD Navigation system: BrainLAB Kolibri ENT system Guidance method for navigation: Z-touch touch laser pointer (BrainLAB AG) Dental impression technique: MD
Kato T., 2023 [15] Stomatol Oral Maxillofac Surg Case Report Included No Funding	Sample size: n.1 Gender ratio: 1M Mean/Range age: 56 y.o. Comorbidities: History of Hepatitis B and hypertension Ongoing pharmacological treatment: MD Dentition status: dentulous	Type: Extraction of third molar (root) Surgery step(s) performed with DCAN: MD Location: mandibular third molar region, tooth root on the sublingual space above the mylohyoid line Other characteristics: bone defect on the lingual side of the extraction socket	Radiographic imaging: CBCT Guidance method for imaging: occlusal splint made with radiopaque resin Planning software: MD Navigation software: None Navigation system: Brainlab Curve ^®^ Guidance method for navigation: resin radiopaque occlusal splint Dental impression technique: MD
Li P., 2015 [27] Int J Oral Maxillofac Surg Case report Included National 863 Program and the Chinese National Natural Science Foundation	Sample size: n.1 Gender ratio: 1F Mean/Range age: 48 y.o. Comorbidities: None Ongoing pharmacological treatment: MD Dentition status: N/D	Type: Removal of foreign body (compound resins used for tooth filling) Surgery step(s) performed with DCAN: minimal abrasion Location: posterior mandible region Other characteristics: proximity to the IAN	Radiographic imaging: CBCT, face scan Guidance method for imaging: MD Planning software: iPlan^®^ Navigation software: BrainLAB^®^ Navigation system: VectorVision2^®^ Guidance method for navigation: self-curing acrylic resin open splint Dental impression technique: MD
Liu J., 2024 [22] Int J Oral Maxillofac Surg Case report Included National Program for Multidisciplinary Cooperative Treatment on Major Diseases and the Program for New Clinical Techniques and Therapies of Peking University School and Hospital of Stomatology	Sample size: N/D Gender ratio: N/D Mean/Range age: N/D Comorbidities: N/D Ongoing pharmacological treatment: N/D Dentition status: partially dentulous	Type: autogenous Bone Ring Technique (bone graft collection) Surgery step(s) performed with DCAN: making an incision at the crest of the ridge, preparing the bone ring bed, embedding the bone ring Location: mandibular branch Other characteristics: N/D	Radiographic imaging: CBCT, digital intraoral scan Guidance method for imaging: N/D Planning software: Mimics Medical 20.0^®^ Navigation software: Digital-care^®^ Navigation system: N/D Guidance method for navigation: corresponded anatomical features of the teeth Dental impression technique: digital impression
Lysenko A., 2022 [36] Imaging Sci Dent Case report Included No Funding	Sample size: n.1 Gender ratio: 1M Mean/Range age: 38 y.o. Comorbidities: MD Ongoing pharmacological treatment: MD Dentition status: dentulous	Type: Removal of cyst Surgery step(s) performed with DCAN: removal of the cyst, apex resection of the medial buccal root of the left upper second molar Location: upper left second and third molar regions Other characteristics: medial buccal root of the left upper second molar is included in one-third of the cyst cavity	Radiographic imaging: CBCT Guidance method for imaging: Radiopaque markers Planning software: MD Navigation software: MD Navigation system: MD Guidance method for navigation: AR glasses, HoloLens Dental impression technique: MD
Maeda K., 2020 [33] Oral Maxillofac Surg Med Pathol Case report Included No Funding	Sample size: n.1 Gender ratio: 1F Mean/Range age: 55 y.o. Comorbidities: None Ongoing pharmacological treatment: MD Dentition status: partially edentulous	Type: Extraction of the tooth root Surgery step(s) performed with DCAN: flap incision, ostectotomy Location: right side of the mandible Other characteristics: proximity to the IAN	Radiographic imaging: CT Guidance method for imaging: MD Planning software: MD Navigation software: MD Navigation system: BrainLAB^®^ Guidance method for navigation: all-in-one splint Dental impression technique: N/D
Magic M., 2020 [21] Int J Oral Maxillofac Implants Case report Included Science and Technology Ministry of China, and Clinical Research Plan of SHDC	Sample size: n.1 Gender ratio: 1F Mean/Range age: 44 y.o. Comorbidities: MD Ongoing pharmacological treatment: MD Dentition status: partially edentulous, severe generalized periodontitis	Type: Osteoplasty before implant placement Surgery step(s) performed with DCAN: osteotomy Location: anterior maxillary region Other characteristics: pneumatization of the maxillary sinuses and severe bone resorption in the posterior maxilla	Radiographic imaging: CBCT, digital intraoral scan Guidance method for imaging: MD Planning software: iPLAN^®^ Navigator BrainLAB^®^ Navigation software: BrainLAB^®^ Navigation system: VectorVision2^®^ Guidance method for navigation: parallel pins Dental impression technique: digital impression
Matsuda S., 2018 [34] J Hard Tissue Biol Clinical report Included No Funding	Sample size: n.6 Gender ratio: 4M/2F Mean/Range age: 64.3 y.o./55–76 y.o. Comorbidities: MD Ongoing pharmacological treatment: MD Dentition status: N/D	Type: Dental implant removal Surgery step(s) performed with DCAN: alveolar bone removal Location: maxillary anterior region (1,2,3), mandibular molar region and maxillary sinus (4), mandibular molar region (5,6) Other characteristics: peri-implantitis (1), fracture of the implant (2,3,5,6), periimplantitis and migration into the maxillary sinus (4), presence of adjacent teeth (1,2,3,4,5)	Radiographic imaging: CBCT Guidance method for imaging: MD Planning software: MD Navigation software: MD Navigation system: MD Guidance method for navigation: reference point fixed on the forehead Dental impression technique: MD
Ohba S., 2014 [20] Odontology Case series Included No Funding	Sample size: n.3 Gender ratio: 2M/1F Mean/Range age: 35.8 y.o./8–66 y.o. Comorbidities: MD Ongoing pharmacological treatment: MD Dentition status: MD	Type: extraction of supernumerary tooth (1), two dental implant screws removal (2), extraction of two supernumerary teeth (3) Surgery step(s) performed with DCAN: N/D Location: upper central region (1,2), region of the mandible (3) Other characteristics: underneath the nasal cavity (1), in contact with the bilateral cortical bone, almost horizontal (2), in proximity of the mental foramen (3)	Radiographic imaging: CBCT (1,3), infrared scanner (1), N/D (2) Guidance method for imaging: splint performed (1,2) Planning software: MD Navigation software: MD Navigation system: iPLAN^®^ Guidance method for navigation: MD (1,2), splint performed (3) Dental impression technique: N/D
Pellegrino G., 2021 [25] Int J Comput Dent Case series Included No Funding	Sample size: n.3 Gender ratio: 2M/1F Mean/Range age: 31.33 y.o./18–51 y.o. Comorbidities: MD Ongoing pharmacological treatment: MD Dentition status: MD	Type: Third molar extraction Surgery step(s) performed with DCAN: circumferential osteotomy of the spongiosa was performed, and odontotomy Location: mandibular third molar region Other characteristics: proximity to the IAN	Radiographic imaging: CBCT Guidance method for imaging: MP Planning software: N/D Navigation software: N/D Navigation system: ImplaNav^®^ Guidance method for navigation: MP keeping attached the patient reference tool Dental impression technique: MD
Retana A., 2019 [35] J Oral Maxillofac Surg Case report Included No Funding	Sample size: n.1 Gender ratio: 1M Mean/Range age: 17 y.o. Comorbidities: MD Ongoing pharmacological treatment: MD Dentition status: dentulous	Type: Extraction of two supernumerary teeth Surgery step(s) performed with DCAN: osteotomies Location: maxillary left central incisor and maxillary right second premolar regions Other characteristics: proximity to maxillary sinus and nasal cavity	Radiographic imaging: CBCT Guidance method for imaging: fiducial apparatus Planning software: MD Navigation software: MD Navigation system: MD Guidance method for navigation: visible fiducial landmarks Dental impression technique: thermoplastic clip adapted to the patient’s dentition as a fiducial apparatus
Stein K. M., 2015 [29] J Oral Maxillofac Surg Case report Included No Funding	Sample size: n.1 Gender ratio: 1F Mean/Range age: 13 y.o. Comorbidities: MD Ongoing pharmacological treatment: MD Dentition status: dentulous	Type: Removal of foreign body (broken dental needle) Surgery step(s) performed with DCAN: flap incision Location: pterygomandibular space Other characteristics: medial to the right mandibular ramus	Radiographic imaging: CBCT Guidance method for imaging: None Planning software: Synergy Fusion ENT 2.2.2^®^ Navigation software: MD Navigation system: Medtronic StealthStation S7^®^ Guidance method for navigation: soft tissue landmarks of the face and hard tissue points with AxiEM Emitter Dental impression technique: None
Sukegawa S., 2017 [28] Med Case Rep Case Report Included No Funding	Sample size: n.1 Gender ratio: 1F Mean/Range age: 65 y.o. Comorbidities: MD Ongoing pharmacological treatment: MD Dentition status: dentulous	Type: Removal of foreign body (dental instrument) Surgery step(s) performed with DCAN: vestibular flap incision, bony window Location: right lower first premolar Other characteristics: proximity to the IAN	Radiographic imaging: OPT, CBCT Guidance method for imaging: interocclusal splint Planning software: Medtronic Navigation Inc.^®^ Navigation software: MD Navigation system: MD Guidance method for navigation: tracker EM for soft tissue landmarks and hard tissue points Dental impression technique: MD
Wang H., 2018 [30] Medicine Case report Included Fundamental Research Fund for the Central Universities of China and the Scientific Rsearch Poject of the Health and Family Planning Commission of Hubei Province	Sample size: n.1 Gender ratio: 1M Mean/Range age: 16 y.o. Comorbidities: MD Ongoing pharmacological treatment: MD Dentition status: dentulous	Type: Extraction of two molars (displaced into the maxillary sinus) Surgery step(s) performed with DCAN: inserting the forceps accurately, clamping the molar one by one, reduction and fixation of the bilateral condyles Location: maxillary sinus Other characteristics: alveolar fracture and gingival laceration in the right maxilla, presence of an orifice fistula, bilateral condyle fractures	Radiographic imaging: OPT, CT Guidance method for imaging: MD Planning software: MD Navigation software: MD Navigation system: VectorVision2^®^ Guidance method for navigation: the surface of face registration by Z-touch laser pointer Dental impression technique: N/D
Wang J., 2017 [37] J Oral Maxillofac Surg Case report Included Beijing Science and Technology Project	Sample size: n.1 Gender ratio: 1M Mean/Range age: 7 y.o. Comorbidities: MD Ongoing pharmacological treatment: MD Dentition status: dentulous	Type: Extraction of two supernumerary Surgery step(s) performed with DCAN: extraction of the two supplementary teeth Location: palatal side of the incisors Other characteristics: The first one is proximal to the nasal floor. The second one is located between an unerupted canine and the immature root of the lateral incisor	Radiographic imaging: CBCT Guidance method for imaging: five radiopaque spheres (BrainLAB^®^) Planning software: iPLAN CMF 2.1 software (BrainLAB^®^) Navigation software: BrainLAB ENT/CMF^®^ Navigation system: N/D Guidance method for navigation: headband and MOR Dental impression technique: MOR
Wang J., 2021 [42] Int J Comput Dent RCT Some concerns Key Projects of International Scientific and Technological Innovation Cooperation, and Beijing Municipal Science & Technology commission	Sample size: n.16 Gender ratio: 15M/1F Mean/Range age: 8 y.o./7–16 y.o. Comorbidities: MD Ongoing pharmacological treatment: MD Dentition status: MD	Type: Extraction of supernumerary Surgery step(s) performed with DCAN: lifting of mucoperiostal flap, opening of the small bony window by using a navigator-tracked electric handpiece, confirming positioning of supplementary teeth Location: palatal side of the incisors Other characteristics: presence of adjacent teeth, nasal floor, maxillary sinus, nasopalatine nerve canal	Radiographic imaging: CBCT Guidance method for imaging: MOR Planning software: iPLAN CMF 2.1 Navigation software: MD Navigation system: Brainlab ENT/CMF^®^ Guidance method for navigation: headband 5 sphered registration points on the MOR and some landmark points added annually on the MOR Dental impression technique: MD
Wu B.Z., 2023 [39] J Dental Sci Prospective analysis Critical risk University School and Hospital of Stomatology	Sample size: n.28 Gender ratio: 15M/13F Mean/Range age: 50 y.o./29–78 y.o. Comorbidities: MD Ongoing pharmacological treatment: MD Dentition status: N/D	Type: TSFE Surgery step(s) performed with DCAN: drilling to reach the panned site, accomplishing the TFSE using a piezoelectric device and osteotomes Location: bottom of maxillary sinus floor Other characteristics: N/D	Radiographic imaging: CBCT Guidance method for imaging: N/D Planning software: Dcarer^®^ Navigation software: Dcarer^®^ Navigation system: N/D Guidance method for navigation: N/D Dental impression technique: digital impression with silicone elastomer
Yamamoto S., 2019 [40] Int J Oral Maxillofac Surg Case report Included No Funding	Sample size: n.1 Gender ratio: 1M Mean/Range age: 31 y.o. Comorbidities: MD Ongoing pharmacological treatment: MD Dentition status: dentulous	Type: Removal of cyst Surgery step(s) performed with DCAN: removal of cyst and surrounding cysts Location: right mandibular second and third molar region Other characteristics: proximity to the IAN	Radiographic imaging: OPT, CBCT Guidance method for imaging: numbered landmarks (10 gutta-percha markers) Planning software: Kick^®^ Navigation software: Kick^®^ navigation system Navigation system: BrainLAB^®^ Guidance method for navigation: numbered landmarks Dental impression technique: conventional impression
Yang C.Y., 2017 [41] Dental Traumatol Case report Included No Funding	Sample size: n.1 Gender ratio: 1F Mean/Range age: 13 y.o. Comorbidities: MD Ongoing pharmacological treatment: MD Dentition status: dentulous	Type: removal of foreign bodies (24 buckshot) Surgery step(s) performed with DCAN: removing through a mini-incision Location: soft tissues of the left buccal sulcus (*n* = 7), of the left labial sulcus (*n* = 3), of the inferior margin of the mandible (*n* = 3), of the submental and submandibular region (*n* = 4), mandible (*n* = 3), floor of the mouth (*n* = 2), neck (*n* = 1), maxillary alveolar mucosa (*n* = 1) Other characteristics: MD	Radiographic imaging: CBCT Guidance method for imaging: buckshot Planning software: MD Navigation software: MD Navigation system: Vector Vision2^®^ navigation system (BrainLAB^®^) Guidance method for navigation: headband and Z-touch laser scanner Dental impression technique: MD
Yang M., 2024 [23] PLoS One Prospective study Serious risk Dengfeng Program of Dalian Stomatological Hospital, China	Sample size: n.35 Gender ratio: 15M/18F Mean/Range age: 46.69 y.o./27–74 y.o. Comorbidities: N/D Ongoing pharmacological treatment: N/D Dentition status: N/D	Type: MSFE (A), TSFE (B) Surgery step(s) performed with DCAN: MD Location: maxilla Other characteristics: 3 mm ≤ RBH < 6 mm (A), 6 mm ≤ RBH < 10 mm (B)	Radiographic imaging: CBCT Guidance method for imaging: MD Planning software: Dcarer^®^ Navigation software: Dcarer^®^ Navigation system: Dcarer^®^ Guidance method for navigation: U-shaped tube Dental impression technique: conventional impression using U-shaped tube placed with polyether (3M ESPE)
Zhang H.X., 2023 [14] J Dentistry Case series Included Program for New Clinical Techniques and Therapies of Peking University School and Hospital of Stomatology	Sample size: n.12 Gender ratio: 5M/7F Mean/Range age: 28.67 y.o./24–32 y.o. Comorbidities: MD Ongoing pharmacological treatment: MD Dentition status: MD	Type: coronectomy of the mandibular third molar Surgery step(s) performed with DCAN: trimming the remaining tooth until it reaches the ideal depth Location: mandibular third molar region Other characteristics: proximity to the inferior alveolar	Radiographic imaging: CBCT, intraoral scan Guidance method for imaging: MD Planning software: GeoMagic^TM^ Studio 12 software^®^ Navigation software: Dcarer^®^ Navigation system: N/D Guidance method for navigation: alignment grooves Dental impression technique: digital impression

Abbreviations: number, “n.”; male, “M”; female, “F”; Dynamic Computer-Aided Navigation, “DCAN”; Inferior Alveolar Nerve, “IAN”; Computer Tomography, “CT”; Missing Data, “MD”; Cone-Beam Computer Tomography, “CBCT”; Standard Tessellation Language, “STL”; Digital Imaging and Communications in Medicine,”DICOM”; Randomized Controlled Trial, “RCT”; Not Defined, “N/D”; Sinus Floor Elevation, “SFE”; Maxillary Sinus Floor Elevation, “MSFE”; Transcrestal Sinus Floor Elevation, “TSFE”; Residual alveolar Bone Height, “RBH”; Marker Plate, ”MP”; Augmented Reality, “AR”; Modified Occlusion Registration, “MOR”; Modified Occlusial Registration “MOR”.

**Table 2:** Extraction data table of the primary and secondary outcomes: primary outcome(s) (angle deviation, entry deviation, depth deviation, linear lateral deviation); secondary outcome(s) (surgical duration, post-operative course, complications type and rate, follow-up, patient- and/or clinician-reported feedback/usability/acceptability/satisfaction).

Studies	Primary Outcome(s)		Secondary Outcome(s)					
	Angle deviation; Entry deviation; Depth deviation; Linear lateral deviation	Other	Surgical duration	Post-operative course	Complications type and rate	Follow-up	Patient-reported feedback/usability/acceptability/satisfaction	Clinician-reported feedback/usability/acceptability/satisfaction
Dentoalveolar Surgeries								
Tooth Extraction								
Maeda 2020 [33] Sample size: n.1	MD	0.5 mm of deviation at the right first mandibular molar	55 min	MD	None	N/D	MD	MD
Wang 2018 [30] Sample size: n.1	MD	MD	50 min	MD	None	At 6 months: no postoperative complications	MD	MD
Extraction of supernumerary tooth								
FangFang 2024 [17] Sample size: n.1	MD	MD	15 min	Mild pain	None	At 2 weeks: pain resolution	Felt good without discomfort	MD
Ohba 2014 [20] Sample size: n.2	MD	MD	MD	MD	MD	MD	MD	MD
Retana 2019 [35] Sample size: n.1	MD	N/D	MD	No erythema, swelling, wound dehiscence, or purulence	None	MD	MD	MD
Wang 2017 [37] Sample size: n.1	MD	Registration accuracy 0.25 mm, accuracy of the landmarks ranged between 0.6–1.4 mm	30 min	No pain, swelling, or bleeding	None	At 3 months: no pain on percussion and normal gingiva	MD	MD
Wang 2021 [42] Sample size: n.16	MD; All access was performed at the planned point; Extra removed bone in length was 0.0 mm (maximum deviation 4.0 mm); Extra removed bone in width was 0.0 mm (maximum deviation 2.0 mm)	MD	About 10 min	Hematoma in one case	Permanent incisor hypoplasia of the root in one case	At 6 months: resolution of the permanent incisor hypoplasia of the root	Pain was measured the second day after surgery using VAS (1.37 ± 0.16)	MD
Extraction of the third molar								
FangFang 2024 [18] Sample size: n.80	MD	MD	37 ± 5 min	MD	None	MD	MD	N/D
Guo 2015 [38] Sample size: n.12	MD	MD	15–30 min	Pain	MD	At 1 week: pain resolution	MD	MD
Kato 2023 [15] Sample size: n.1	MD	MD	MD	MD	MD	MD	MD	MD
Pellegrino 2021 [25] Sample size: n.3	MD	MD	20 min	MD	None	None	MD	Enabled root fork identification and complete root separation
Coronectomy of the third molar								
Zhang 2023 [14] Sample size: n.12	MD	Root mean square deviation: 0.69 ± 0.21 mm (maximum 1.45± 0.83/1.87 ± 0.63)	30–40 min	MD	None	At 3 months: no infections, pulpitis, dry socket, post operative root eruption	MD	Navigation system is not equipped to support irregular 3D boundaries for performing tooth sectioning
Removal of foreign bodies								
Chen 2020 [31] Sample size: n.1	MD	MD	MD	MD	MD	MD	MD	MD
Chen 2019 [24] Sample size: n.1	MD	Registration accuracy 0.8 mm	60 min	MD	None	At 1 month: satisfactory wound healing and mouth opening, without postoperative complications	MD	MD
Li 2015 [27] Sample size: n.1	MD	Registration accuracy 0.8 mm	20 min	MD	None	None	MD	MD
Stein 2015 [29] Sample size: n.1	MD	MD	15 min	MD	None	A few weeks: normal range of motion	MD	MD
Sukegawa 2017 [28] Sample size: n.1	MD	MD	MD	MD	MD	MD	MD	MD
Yang 2017 [41] Sample size: n.1	MD	MD	MD	MD	None	Satisfactory wound healing and mouth opening	MD	MD
Dental implant removal surgeries								
Matsuda 2018 [34] Sample size: n.6	MD	MD	MD	MD	None	N/D	MD	MD
Ohba 2014 [20] Sample size: n.1	MD	MD	MD	MD	MD	MD	MD	MD
Cyst removal								
Lysenko 2022 [36] Sample size: n.1	MD	Root mean square: between 3–6 mm	MD	MD	None	MD	MD	MD
Yamamoto 2019 [40] Sample size: n.1	MD	MD	MD	MD	MD	MD	MD	MD
Removal of bone graft fixing screws								
Casap 2006 [32] Sample size: n.1	MD	MD	N/D	MD	MD	MD	MD	MD
Sequestrectomy at stage 2 of Medication-Related Osteonecrosis of the Jaws								
Chen 2020 [16] Sample size: n.1	MD	MD	MD	MD	None	At 1 month: lip dysesthesia resolution	MD	Device overcame the issue of the mandible mobility
Osteoplasty before the implant placement								
Magic 2020 [21] Sample size: n.1	N/D	Mean deviation: 1.3 ± 0.39 mm (from 0.8 to 1.7 mm)	MD	MD	MD	MD	MD	MD
Bone Augmentation surgeries								
Sinus Elevation Surgeries								
Dotia 2024 [19] Sample size: n.1	8.93°; 2.83 mm; 0.29 mm; 2.52 mm	MD	MD	MD	MD	MD	MD	Low accuracy to identify sinus membrane from the overall soft tissues
Wu 2023 [39] Sample size: n.28	N/D	N/D	MD	MD	None	MD	MD	MD
Yang 2024 [23] Sample size: n.35	N/D	N/D	MD	MD	None	N/D	MD	MD
“Sandwich” procedure								
Felice 2021 [26] Sample size: n.1	MD	MD	MD	MD	None	2 weeks and 1 month	MD	MD
Autogenous Bone Ring Technique								
Liu 2024 [22] Sample size: N/D	N/D	MD	MD	MD	None	N/D	MD	MD

Abbreviations: number, “n.”; minutes, “min”; millimeters, “mm”; Missing Data, “MD”; Not Defined, “N/D”; three-dimensional, “3D”.

## Data Availability

Data are available in the MEDLINE/PubMed, Web of Science, Scopus, and Cochrane Library databases, and in the PROSPERO register.

## Supplementary Materials

The following supporting information can be downloaded at https://www.mdpi.com/article/10.3390/healthcare13141730/s1, File S1: Full Search Strategy; File S2: Quality Assessment.
