# Supplementary material for: Dynamic Computer-Aided Navigation System in Dentoalveolar Surgery and Maxillary Bone Augmentation in a Dental Setting: A Systematic Review
Source: Healthcare (Basel). 2025 Jul 17;13(14):1730. doi: 10.3390/healthcare13141730 (PMC12296081; doi:10.3390/healthcare13141730)
Supplement: Supplementary file 1 [file healthcare-13-01730-s001.zip › Navigation Full Search Strategy - Supplementary File S1.pdf]

SUPPLEMENTARY FILE S1 – FULL SEARCH STRATEGY

Table S1. Full search strategy of each database searched.

Database	Date of Search	String	Filters applied
PubMed/MEDLINE	28 October 2024	((("dynamer"[All Fields] OR "dynamers"[All Fields] OR "dynamic"[All Fields] OR "dynamical"[All Fields] OR "dynamically"[All Fields] OR "dynamicity"[All Fields] OR "dynamics"[All Fields] OR "dynamism"[All Fields] OR "dynamisms"[All Fields]) AND ("surgery, computer assisted"[MeSH Terms] OR ("surgery"[All Fields] AND "computer assisted"[All Fields]) OR "computer-assisted surgery"[All Fields] OR ("computer"[All Fields] AND "aided"[All Fields] AND "surgery"[All Fields]) OR "computer aided surgery"[All Fields])) OR "real-time navigation"[All Fields] OR "real-time surgery"[All Fields] OR "navigation system"[All Fields] OR "dynamic guided surgery"[All Fields] OR "dynamic computer-assisted surgery"[All Fields] OR "dynamic navigation"[All Fields] OR "navigation surgery"[All Fields]) AND ("oral surgery"[All Fields] OR ("dentistry"[MeSH Terms] OR "dentistry"[All Fields] OR "dentistry s"[All Fields])) AND ("dynamer"[All Fields] OR "dynamers"[All Fields] OR "dynamic"[All Fields] OR "dynamical"[All Fields] OR "dynamically"[All Fields] OR "dynamicity"[All Fields] OR "dynamics"[All Fields] OR "dynamism"[All Fields] OR "dynamisms"[All Fields] OR ("navigability"[All Fields] OR "navigable"[All Fields] OR "navigate"[All Fields] OR "navigated"[All Fields] OR "navigates"[All Fields] OR "navigating"[All Fields] OR "navigation"[All Fields] OR "navigational"[All Fields] OR "navigations"[All Fields] OR "navigator"[All Fields] OR "navigator s"[All Fields] OR "navigators"[All Fields]))	English
Scopus	28 October 2024	TITLE-ABS-KEY (("dynamic computer-aided surgery" OR "real-time navigation" OR "real-time surgery" OR "navigation system" OR "dynamic guided surgery" OR "dynamic computer-assisted surgery" OR "dynamic navigation" OR "navigation surgery") AND ("oral surgery" OR dentistry) AND (dynamic OR navigation))	English
Web of Science	28 October 2024	ALL=(("dynamic computer-aided surgery" OR "real-time navigation" OR "real-time surgery" OR "navigation system" OR "dynamic guided surgery" OR "dynamic computer-assisted surgery" OR "dynamic navigation" OR "navigation surgery") AND ("oral surgery" OR dentistry) AND (dynamic OR navigation) (All Fields))	English
Cochrane Library	28 October 2024	(("dynamic computer-aided surgery" OR "real-time navigation" OR "real-time surgery" OR "navigation system" OR "dynamic guided surgery" OR "dynamic computer-assisted surgery" OR "dynamic navigation" OR "navigation surgery") AND ("oral surgery" OR dentistry) AND (dynamic OR navigation)):ti,ab,kw	English
