# Supplementary material for: Dynamic Computer-Aided Navigation System in Dentoalveolar Surgery and Maxillary Bone Augmentation in a Dental Setting: A Systematic Review
Source: Healthcare (Basel). 2025 Jul 17;13(14):1730. doi: 10.3390/healthcare13141730 (PMC12296081; doi:10.3390/healthcare13141730)
Supplement: Supplementary file 1 [file healthcare-13-01730-s001.zip › Navigation Quality Assessment - Supplmentary file S2.pdf]

SUPPLEMENTARY FILE S2 - QUALITY ASSESSMENT

Table S2. Quality assessment of included case reports (sorted alphabetically) according to the Johanna Briggs Institute Critical Appraisal Checklist for case reports. Abbreviations: "Y", yes; "N", no; "U", unclear.

Study	Item 1	Item 2	Item 3	Item 4	Item 5	Item 6	Item 7	Item 8
**Casap et al.,** 2006 [32]	N	Y	Y	Y	Y	N	N	Y
**Chen et al.,** 2019 [24]	N	N	Y	Y	Y	U	N	Y
**Chen et al.,** 2020 [31]	N	N	Y	Y	Y	N	U	Y
**Chen et al.,** 2020 [16]	N	Y	Y	Y	Y	Y	Y	U
**Dotia et al.,** 2024 [19]	N	N	Y	Y	Y	N	N	Y
**FangFang et al.,** 2024 [17]	N	N	Y	Y	Y	Y	Y	Y
**Felice et al.,** 2021 [26]	N	Y	Y	Y	Y	Y	N	Y
**Kato et al.,** 2023 [15]	N	Y	Y	Y	Y	U	N	Y
**Li et al.** 2015 [27]	N	Y	Y	Y	Y	N	N	Y
**Liu et al.** 2024 [22]	N	Y	N	Y	Y	N	N	U
**Lysenko et al.** 2022 [36]	N	Y	Y	U	Y	N	N	Y
**Maeda et al.,** 2020 [33]	N	Y	Y	Y	Y	Y	N	Y
**Magic et al.,** 2020 [21]	N	Y	Y	Y	Y	U	N	Y
**Retana et al.,** 2019 [35]	N	Y	Y	Y	Y	Y	N	Y
**Stein et al.,** 2015 [29]	N	Y	Y	Y	Y	U	N	Y
**Sukegawa et al.,** 2017 [28]	N	Y	Y	Y	Y	Y	N	Y
**Wang et al.,** 2017 [37]	N	U	Y	Y	Y	Y	N	Y
**Wang et al.,** 2018 [30]	N	Y	Y	Y	Y	U	N	Y
**Yamamoto et al.,** 2019 [40]	N	Y	Y	Y	Y	N	U	Y
**Yang et al.,** 2017 [41]	N	Y	Y	Y	Y	U	N	N

Table S3. Quality assessment of included case series (sorted alphabetically) according to the Johanna Briggs Institute Critical Appraisal Checklist for case series. Abbreviations: "Y", yes; "N", no; "U", unclear; "N.A.", Not Applicable.

Study	Item 1	Item 2	Item 3	Item 4	Item 5	Item 6	Item 7	Item 8	Item 9	Item 10
**Guo et al.,** 2015 [38]	Y	Y	Y	N	Y	U	Y	Y	Y	U
**Matsuda et al.,** 2018 [34]	Y	Y	U	N	Y	U	Y	Y	Y	Y
**Ohba et al.,** 2014 [20]	N	U	U	U	U	N	Y	Y	Y	N.A.
**Pellegrino et al.,** 2021 [25]	Y	Y	Y	N	Y	U	Y	Y	Y	Y
**Zhang et al.** 2023 [14]	Y	U	U	N	Y	N	U	U	U	Y

Table S4. Quality assessment of included randomized controlled trials (sorted alphabetically) according to the revised Cochrane Risk of Bias for Randomized (RoB-II) Studies of Interventions.
Abbreviations: "Y", yes; "PY", Probably yes"; "N", no; "PN", Probably No; "NA", Not Applicable; "NI", No Information".

	**Randomization process (item 1)**			**Effect of assignment to intervention (item 2.a)**							**Effect of adhering to intervention (item 2.b)**						**Missing outcome data (item 3)**				**Measurement of the outcome (item 4)**					**Selection of the reported studies (item 5)**		
	1.1	1.2	1.3	2.1	2.2	2.3	2.4	2.5	2.6	2.7	2.1	2.2	2.3	2.4	2.5	2.6	3.1	3.2	3.3	3.4	4.1	4.2	4.3	4.4	4.5	5.1	5.2	5.3
**FangFang et al.,** 2024 [18]	Y	Y	N	PY	Y	N	NA	NA	Y	NA	PY	Y	Y	N	N	NA	Y	NA	NA	NA	PN	PN	Y	N	NA	Y	N	N
Overall risk of bias: Low Risk	Low Risk			Low Risk							Low Risk						Low Risk				Low Risk					Low Risk		
**Wang et al.,** 2021 [42]	Y	Y	N	Y	Y	PN	NA	NA	PY	NA	Y	Y	NI	N	PN	Y	Y	NA	NA	NA	N	N	Y	N	NA	Y	N	N
Overall risk of bias: Some concerns	Low Risk			Low Risk							Some concerns						Low Risk				Low Risk					Low Risk		

Table S5. Quality assessment of included non-randomized studies (sorted alphabetically) according to the Risk of Bias for non-Randomized Studies of Interventions (ROBINS-I).
Abbreviations: "Y", yes; "PY", Probably yes"; "N", no; "PN", Probably No; "NA", Not Applicable; "NI", No Information".

**Item**	**Wu et al.,** 2022 [39]	**Yang at al.,** 2024 [23]
**Bias due to confounding**		
1.1	PY	PY
1.2	PN	N
1.3	NA	NA
1.4	PN	PY
1.5	NA	Y
1.6	PN	PN
1.7	PN	NA
1.8	PN	NA
	Critical risk	Moderate risk
**Bias in selection of participants into the study**		
2.1	Y	Y
2.2	PY	PY
2.3	PY	PY
2.4	Y	Y
2.5	PN	PN

	Serious risk	Serious risk
**Bias in classification of interventions**		
3.1	N	Y
3.2	PY	PY
3.3	PN	PN
	Serious risk	Moderate risk
**Bias due to deviations from intended interventions**		
4.1	N	N
4.2	NA	NA
4.3	Y	Y
4.4	Y	Y
4.5	Y	Y
4.6	NA	NA
	Low risk	Low risk
**Bias due to missing data**		
5.1	PY	Y
5.2	NI	N
5.3	NI	N
5.4	NA	NA
5.5	NA	NA
	No information	Low risk
**Bias in measurement of outcomes**		
6.1	N	N
6.2	PN	Y
6.3	Y	Y
6.4	N	NI
	Low risk	Moderate risk
**Bias in selection of the reported result**		
7.1	N	N
7.2	N	N
7.3	N	N
	Low risk	Low risk
**Overall risk of bias**	Critical risk	Serious risk
